# Spatial analyses of archaeobotanical record reveal site uses and activities at Early to Middle Holocene Takarkori (Libya, Central Sahara)

**DOI:** 10.1371/journal.pone.0310739

**Published:** 2024-10-23

**Authors:** Savino di Lernia, Fabrizio Buldrini, Assunta Florenzano, Anna Maria Mercuri, Varinia Nardi, Rocco Rotunno

**Affiliations:** 1 Dipartimento di Scienze dell’Antichità, Sapienza University of Rome, Rome, Italy; 2 GAES, University of Witwatersrand, Johannesburg, South Africa; 3 Dipartimento di Scienze Biologiche, Geologiche e Ambientali, Università di Bologna, Bologna, Italy; 4 Laboratorio di Palinologia e Paleobotanica, Dipartimento di Scienze della Vita, Università di Modena e Reggio Emilia, Modena, Italy; 5 The Archaeological Mission in the Sahara, Sapienza University of Rome, Rome, Italy; University of Michigan, UNITED STATES OF AMERICA

## Abstract

This study investigates botanical remains from the Takarkori site in the Tadrart Acacus region (SW Libya) to reconstruct socio-economic and cultural characteristics of human groups during the Holocene. By analyzing micro- and macrofossils of plant origin, we aim to understand the availability and management of environmental resources and how plant taxa were used by humans. The exceptional preservation of archaeobotanical material across all occupation levels, facilitated by the region’s geomorphological and environmental conditions, provides a unique opportunity to study pre-Pastoral and Pastoral Neolithic activities within a comprehensive diachronic framework. Our research extends previous investigations by examining the spatial distribution of archaeobotanical remains in association with site furniture and material correlates, offering insights into the functional use of space within the site. Also, the features of plant assemblages and their distribution patterns indicate the planning in the use of plant resources and the diverse uses beyond subsistence, including ritual and cultural practices. The findings contribute to a deeper understanding of Holocene environmental and cultural dynamics, highlighting the importance of archaeobotanical data in archaeological research.

## Introduction

The complex relationship between humans and plants has been widely addressed in archaeological research. Plant remains as macrofossils and pollen provide crucial insights into the subsistence strategies and land use patterns of ancient communities [e.g. 1–4] Archaeobotany, also including the analyses of phytoliths, starch grains, lipids and other molecules, has emerged as a vital field for identifying activity areas in prehistoric habitation sites [e.g. 5–8]. By analyzing charred seeds, wood, pollen and other plant remains, researchers infer past dietary preferences, and the use of plants for medicinal, ritual, and construction purposes [9]. The examination of remains from cultivated crops, along with those from wild plants, and the reconstruction of agricultural practices, offer essential insights into the extent of agricultural activities, innovations in farming, and the level of sedentism within ancient communities. Moreover, the study of pollen and phytoliths reveal changes in vegetation, land use, and environmental conditions, contributing to our understanding of past ecosystem dynamics and their interplay with human activities [10–13]. Spatial analysis techniques provide a powerful toolkit for investigating the distribution, density, and arrangement of archaeological contexts and features, including plant remains [e.g. 14]. Geographic Information Systems (GIS) and geospatial analysis methods offer efficient means to map, analyze, and interpret spatial data related to habitation sites [15–17]. These techniques enable researchers to identify settlement patterns, recognize activity areas, and uncover patterns of land use and resource exploitation.

By integrating spatial analysis with archaeological and archaeobotanical data, researchers can discern the functional zoning of prehistoric settlements, identify areas of intensive activities such as food processing, craft production, and communal spaces, and explore the socio-economic organization within the archaeological sites [18, 19]. Furthermore, the combination of archaeobotany and spatial analysis facilitates the identification of subtle site boundaries, transitional zones, and the detection of hidden or ephemeral features that might be overlooked by traditional excavation methods alone [20]. The main objective of this work is to apply these interdisciplinary approaches to the Takarkori site, one of the most important archives of Holocene Saharan prehistoric societies [21], aiming to extract comprehensive environmental and cultural information from the spatial distribution of botanical artefacts. This research will contribute to a deeper understanding of Holocene Saharan communities, how they organized their activities spatially within the site, and their adaptive strategies during changing and varying environmental and climate settings.

## The context

Located in south-western Libya, the Takarkori rock shelter opens on the western slopes of the Tadrart Acacus, a massif in the central Sahara. The shelter looks to the west and extends for about 80 m in a north-south direction on a wide terrace, bordered to the east by a rock cliff about 30 m high [21]. The region is currently characterized by hyper-arid climate with low rainfall and vegetation limited to desert savannah with specialized species, such as acacia (*Vachellia tortilis* (Forssk.) Galasso & Banfi subsp. *raddiana* (Savi) Kyal. & Boatwr., formerly named *Acacia tortilis* Forssk. subsp. *raddiana* (Savi) Brenan) and tamarisk (*Tamarix aphylla* (L.) H.Karst.) usually close to synanthropic areas [22].

**Fig 1:**
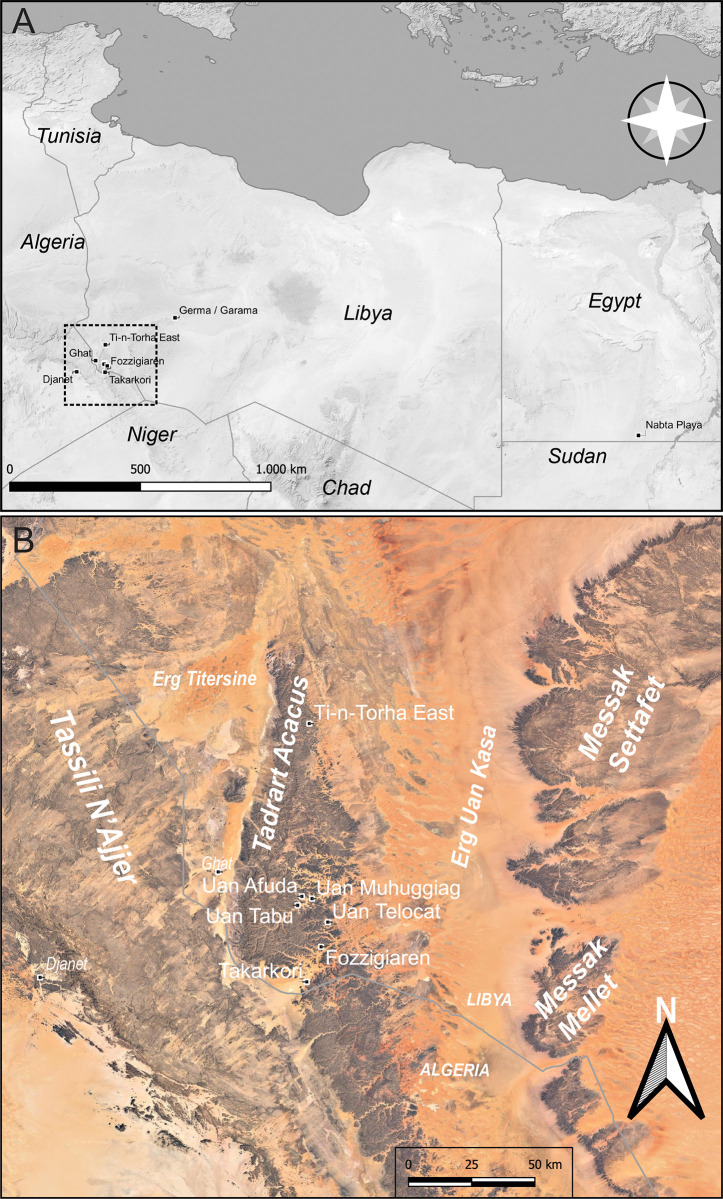
Study area. A) Map of main localities cited in the text; dashed rectangle indicates (B) the study area in southwestern Libya (Background images from ESRI and OPM).

From a geomorphological point of view [23], this area can be defined as a vast geosyncline characterized by large rock formations (Tadrart Acacus massif and Messak plateau), large dune fields (Erg Titersine, Uan Kasa, and Edeyen of Murzuq), valleys and fossil river networks locally termed ‘wadi’ (Wadi Takarkori, Wadi Tanezzuft, and Wadi Barjuj). The Tadrart Acacus massif extends approximately 150 km north-south (26–24° North), near the current border between Libya and Algeria (Fig 1). From an orographic point of view, this formation is similar to the vast mountainous complex of the Algerian Tassili-n-Ajjer, from which it is separated by the Wadi Tanezzuft to the west, while the vast expanses of the Erg Titersine and Uan Kasa encircle its northwestern and eastern limits respectively [23, 24]. The Wadi Takarkori separates the Tadrart Acacus to the north from the Algerian Tadrart to the south.

**Fig 2:**
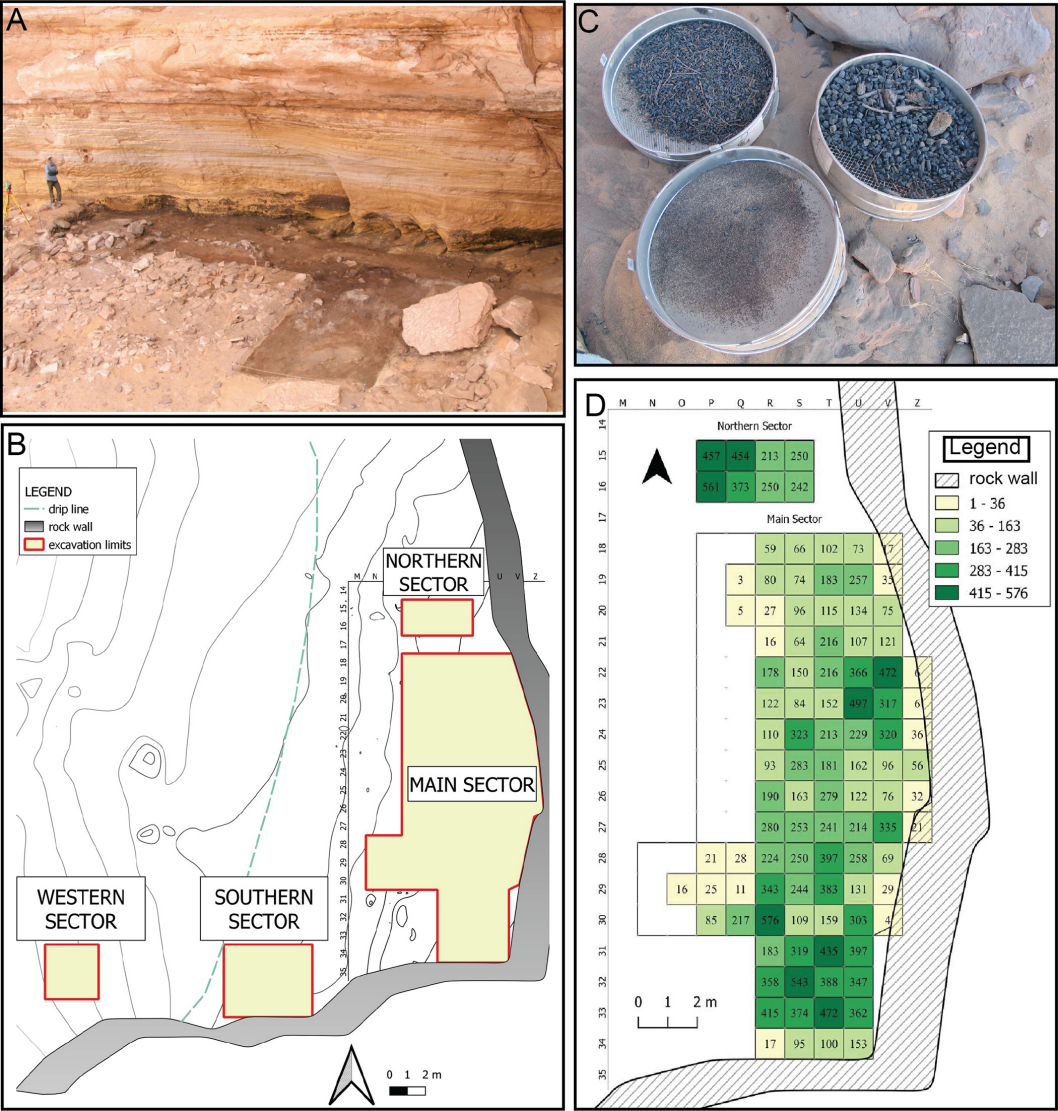
The context and sampling. A) a view of the rock shelter from south-west; B) site topography and excavation sectors; C) 5 l volumetric sampling: the meshes used for dry sieving in the field (decreasing width: 10, 2 and 0.5 mm); D) sampling strategy with relative and absolute frequency of archaeobotanical remains retrieved from sampling evidencing absence of documentation/recording gaps (photos ©The Archaeological Mission in the Sahara).

The rock shelter (Fig 2A) was investigated as part of the larger Wadi Takarkori Project [25, 26], a territorial research program in the southern Tadrart Acacus. The investigations were carried out through four excavation campaigns, from 2003 to 2006, while subsequent activities on the site were interrupted due to geopolitical events linked to the ‘Arab Spring’ [27]. The archaeological sequence of the Takarkori shelter encompasses over 5000 years, from the occupation of hunter-gatherer-fishers locally called Late Acacus, to the Early, Middle and Late phases of the Pastoral Neolithic. The stratigraphic excavation, secured by a significant number of ^14^C dates [28], led to the definition of additional sub-phases (Table 1). Early Holocene use (Late Acacus (LA) phase) was by hunter-gatherer-fishers, followed by a prolonged Pastoral Neolithic occupation (Early Pastoral (EP), Middle Pastoral (MP), Late Pastoral (LP) phases) marked by cattle and ovicaprine herders in the Middle and Late Holocene. Stone structures, fireplaces, grinding stones, and potsherds indicate semi-residential utilization by LA dwellers [29]. Palaeoenvironmental and palaeovegetational reconstructions indicate grasslands and sparse wooded savannahs dominated, along with permanent freshwater habitats characterized by pondweeds and reeds [25], and a progressively more unstable climatic context towards its ending, culminating with an arid phase centered around the so-called 8.2 ka “event” [30]. The EP phase displays fireplaces, stone structures, and human graves. The environment was warmer, where wet habitats diminished while dry shrubs and herbs, mainly belonging to the Asteraceae family and Chenopodioideae sub-family, expanded. The MP phase highlights a cattle-focused economy with evidence of dairying and saw increasing aridity and the spread of xerophytes like acacias. Lastly, organic remains, cuvette hearths, and ovicaprine dung point to specialized LP goat herders adapted to extremely dry conditions and desert savannah.

**Table 1:** Main socio-cultural phases and eco-climatic features.

Cultural Phase	Sub-phase	uncal BP	cal BP	Socio-cultural features	Eco-climatic features
Late Acacus (LA)	LA1	8900–8500	10170–9400	Hunter-gatherer-fishers	Wet savannah with permanent water reservoirs, grasslands and sparse tree cover
	LA2	8500–7900	9500–8600		
	LA3	7900–7400	9000–8000		
Early Pastoral (EP)	EP1	7400–6900	8300–7600	Early herders	Increasing aridity with seasonality. Grassland cover of shrub-steppe and savannah
	EP2	6900–6400	7800–7300		
Middle Pastoral (MP)	MP1	6100–5500	7100–6200	Mature Neolithic pastoralism	Strong seasonality with wet and dry spells, grasslands, rare tree cover and shrub-steppe
	MP2	5500–5000	6400–5600		
Late Pastoral (LP)	LP1	5000–4000	5900–4300	Specialized nomadic herders	Hyperarid desert savannah with *Vachellia* and *Tamarix* associations

Main socio-cultural phases and eco-climatic features identified in the Takarkori area (modified, after [25, 28]). The calibrated dates express the maximum chronological range and overlaps are statistically possible. For the calibration: OxCal online version 4.4.4 [31].

## Data collection and methods

### The excavations

The stratigraphic excavations were organized into four different sectors (Fig 2B), using a 1x1 m grid set up on the entire area of the terrace (2200 m^2^). The total excavated area is 143 m^2^. The Main Sector (MS therein) with 117 m^2^ is the largest area of investigation: here the excavation was interrupted for conservation purposes. The bedrock was reached instead in the Northern Sector (NS therein), an 8 m^2^ trench, in which the oldest occupation levels, related to LA1, were identified immediately lying on the bedrock, ~1.7 m below the present surface. The Southern Sector (SS therein) covers an area of 20 m^2^ including a conical tumulus (T1), that did not yield any human remains. The Western Sector (WS), a 9 m^2^ trench, is the only excavated area beyond the rain dripline, outside of the vault cover. Due to their location outside the drip line of the shelter and being affected by various natural and anthropogenic formation processes, the deposits in SS and WS do not provide a clear stratigraphy allowing a solid archaeobotanical sampling [21, 25]. Therefore, this paper reports on the distribution pattern of the archaeobotanical record from the MS and NS sectors.

### Categories of Archaeological Contexts (ACs)

The classification system adopted here, describing the unique characteristics of archaeological deposits in arid environments, considers the physical and spatial characteristics of the context, the nature of the sediment, as well as the most frequent organic and inorganic components (Table 2). It includes four main categories of Archaeological Contexts (ACs) proposed by [21]:

- *Matrices*: “the sedimentary by-products of human occupation, consisting of organic matter (ovicaprid excrement, plant macromaterials, faunal specimens, etc.) mixed with sands”;
- *Fixtures*: “a large category, highly diversified, and with different functional meanings (…), the Fixtures are the clear outcome of deliberate human intervention and are characterized by an unambiguous functional attribution”;
- *Burials*: the rock shelter yielded the remains of 15 individuals of women and children [26]. The burials belong to different cultures, mainly of Pastoral age;
- *Physiogenetic contexts*: these layers mainly consist of surface aeolian sand, collapsed rocks, altered bedrock.

**Table 2:** Main features of the Archaeological Contexts (ACs) at Takarkori.

Category of AC	Type of AC	Physical state		Sediment	
		spatial configuration	spatial consistency	hardness	texture
*Matrixes*	M1—coarse sand	diffuse	low	very low	poorly sorted
	M2—humified organic sand	diffuse	low	low	well sorted
	M3—cemented organic sand	clear	high	very high	sorted
	M4—organic sand	diffuse	low	low	sorted
	M5—stone accumulation	diffuse	high	low	well sorted
	M6—plant accumulation	clear	high	low	poorly sorted
	M7—dung	diffuse	high	high	sorted
	M8—floor	diffuse	high	high	sorted
*Fixtures*	F1—pit (filling)	clear	high	low	poorly sorted
	F2—post hole (filling)	clear	high	low	poorly sorted
	F3—kettle	clear	high	very high	poorly sorted
	F4—hearth	clear	high	low	sorted
	F5—ash dump	clear	high	high	well sorted
	F6—informal stone structure	clear	low	-	-
	F7—formal stone structure	clear	high	-	-
*Burials*	B—burial	clear	high	-	-
*Physiogenetic*	Ph1—aeolian ingression	diffuse	low	very low	poorly sorted
	Ph2—vault/wall collapse; altered bedrock	clear	low	-	-

Spatial configuration refers to the visibility/readability of ACs’ limits; spatial consistency defines the uniformity/homogeneity of sediment (modified, after [21]).

### Sampling for plant macroremains

The plant materials were collected using three different types of sampling, named as follows:

- *Sieve*: sieving of all the excavated sediment from each stratigraphic unit using a 4-mm-diameter mesh.
- *Volumetric*: sieving standard sediment volumes (5 liters) taken from each stratigraphic unit, with a series of three overlapping meshes of decreasing diameter (10, 2 and 0.5 mm) (Fig 2C).
- *Spot*: direct collection of particularly significant materials which include different ecofacts. Moreover, they also include artifacts and relevant structural elements such as plant fiber weavings or wooden poles. These ‘spot’ samples were systematically identified with a unique code (ID) and recorded by Electronic Total Station (ETS).

For the purpose of this study the plant remains from Sieve and Volumetric sampling are primarily included in statistical elaborations, while Spot sampling of accumulations of Seeds [4], and handpicked Basketry/Cordage remains [32] are considered only as additional elements for the analysis of the spatial layout. Seeds coming from Sieve and Volumetric sampling are considered when useful for the general interpretation and represent additional and unpublished material.

All excavation squares were included in the sample collection (Fig 2D), confirming the reliability of the sampling strategy.

### Plant macroremain extraction and analysis

A systematic layer-by-layer sampling was conducted at the site, with a total number of at least 2000 samples collected in the field. Among them, 1112 samples were analyzed in detail for their rich content in plant macroremains. Ecofacts and artifacts were manually extracted from each sample, weighed with a digital scale, and measured in length with a digital calipers Mitutoyo Digimatic CD– 15D. The state of preservation was evaluated through a 5-step qualitative scale (1: bad, 2: poor, 3: sufficient, 4: good, 5: excellent; from hardly identifiable to very well preserved–mummified–record). Plant macroremains were observed with a stereomicroscope Leica Wild M10 (magnification of 25x to 80x). Carpological and xylo-anthracological atlases, keys and reference collections were used to identify remains with diagnostic characters [1, 19, 33–40]. Charcoal and wood ecofacts were identified through the analysis of wood anatomy, observed on cross and longitudinal sections, or along fresh, hand-made fractures.

### Intra-site spatial analysis

Intrasite spatial analyses aim at investigating patterns and relationships within and between small units of observations across time and space [8, 17, 41]. We analyze here the spatial distribution of archaeobotanical remains within several occupation horizons employing spatial statistical methods within the range of Point Pattern Analyses (PPA) [8, 42, 43], via native algorithms both in R, and additional plugins in the QGIS environment (ver. 3.16.16).

For each occupation sub-phase and material class category selected (see further), we performed a chi-squared test of complete spatial randomness (CSR) using quadrat counts test (QCT) methods to assess the spatial pattering of each observation [14, 44].

To understand the directionality of the pattern evidenced by QCT (i.e., if the deviation from the CSR is driven either by a clustering or dispersion pattern), we performed a distance-based Point Pattern Analysis (PPA). For each sub-phase and each material category we performed a Monte Carlo test for the point pattern. It generates randomly distributed points within the study region and uses the Ripley’s K function for each set of generated simulated points (100 iterations) and compares them to the K-function for the original set of points to determine whether the point pattern deviates at a given distance from complete spatial randomness (CSR) as null hypothesis.

The “spatstat” library [45] and “maptools” library in R (R Core team 2022) were used via the QGis plugin interface as R dependencies, which allows the performance of statistical analyses of spatial point patterns (SPPs) represented in 2D providing data on the intensity and type of the pattern and the relationships among and within different types of point [45, 46].

To assess the actual location of the identified spatial pattern within the site, the spatial analysis of the botanical record was explored out using the “Kernel Density Estimation” (KDE) plugin. It is a spatial interpolation technique that provides an estimate of the density of points in a hypothetical continuous surface, producing clusters or areas with a high density of events. This surface is obtained by cumulating the density functions for individual events, considering for each of them a bandwidth (radius *r* of predefined value). In this study, we chose a radius of *r* = 1 m. This selection considers the sampling strategy and the source of the materials used. These plant remains mainly result from sifted sediment from each stratigraphic unit and are associated with the centroid of the corresponding square. The reference values are represented by a chromatic gradient that expresses density curves defined by the *k* function, which gives a weight to the points that fall within the search radius [47, 48]. In this way, quality concentration maps (heatmaps) are produced, which allow us to visually ascertain the distributional trend of the materials and their interaction both with other plant categories that make up the record and with the main archaeological structures.

Given the palimpsest nature of the site and its pluri-stratigraphic layout, we consider here materials assigned to each sub-phase as pertaining to a coarsely contemporaneous occupation phase. Though inherent issues related to time-average phenomena, such approach has been widely adopted in intra-site analysis for archaeological interpretation and supported by influential research [49–53].

Where spatial analysis revealed potentially significant distributions, the degree of association between the specific categories of material was statistically measured by calculating Pearson’s correlation coefficient *r*. The analysis was carried out with Past© software (v. 4.03).

## Results: The archaeobotanical record

The Takarkori archaeobotanical record here considered consists of a total of 20,540 specimens of different nature (Table 3: 99% from ‘Sieve’ and “Volumetric” sampling, and 1% from ‘Spot’ samples).

**Table 3:** Plant ecofacts and artifacts, per category and type of sampling.

Class	Category	Sieve/Volumetric	Spot*	Number of specimens
**Ecofacts**	Charcoals	7155	2	7157
	*Barks*	5328	3	5331
	Grass stems	1297	2	1299
	Leaves	7	-	7
	Wooden *Sticks*	1832	22	1854
	Roots	61	-	61
	*Twigs*	2021	-	2021
	Thorns	24	-	24
	Inflorescences	10	1	11
	*Fruits*	2631	1	2632
	Seeds	22	30**	22
*Total*		*20388*	*31*	*20419*
**Artifacts**	Cordage	*-*	38	38
	Basketry	4	49	53
	Wooden tool	*-*	25	25
	Wooden pole	*-*	5	5
*Total*		*4*	*117*	*121*
***Grand total***				***20540***

*spot samples of handpicked Basketry and Cordage remains [31]and accumulation of Seeds [4] are discussed elsewhere.

**number of specimens in Spot of Seed accumulations are not counted here, but available in [4].

Eleven categories of *ecofacts* and 4 categories of *artifacts* were identified (Table 3).

### Ecofacts

**Fig 3:**
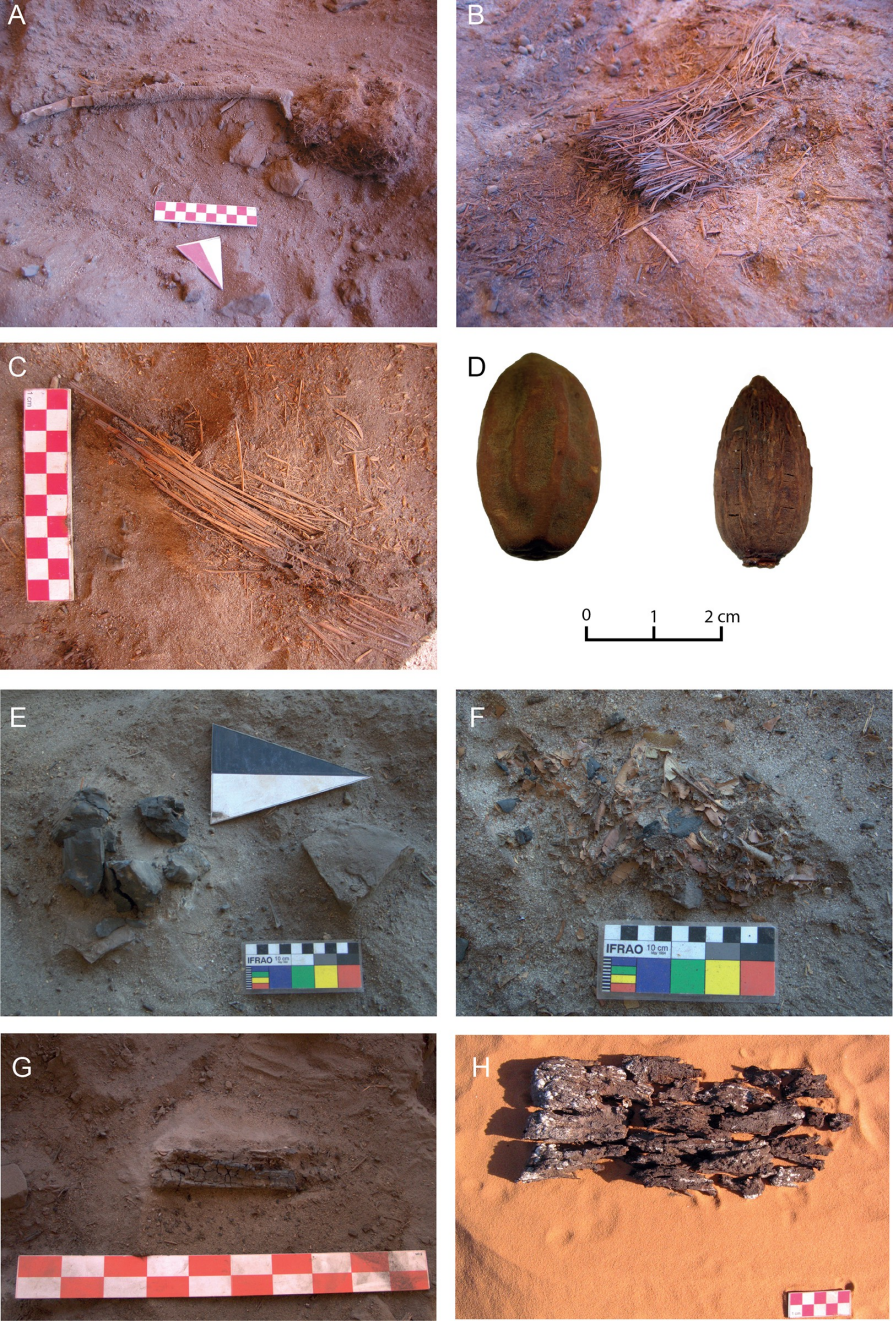
Some examples of ecofacts and chronology. A) Twig (MP2); B) Bundle of grass partially burned on one side (LA3); C) Stems and sticks (LA2); D) Intact *Balanites aegyptiaca* (L.) Delile fruit (LP1) and endocarp (LA3); E) Charcoals from a fireplace (MP2); F) Leaves still in place (MP2); G) (LA3) and H) (EP1) Wood remains with bark (photos ©The Archaeological Mission in the Sahara).

“Ecofacts” include a total of 20,419 elements (excluding samples from Seed Accumulations [4]) that, based on their different botanical nature, were divided in the following 11 categories (Fig 3):

- *Charcoals*: loose, scattered fragments of charred wood (size ≥ 0.5 cm). In most cases, they were fragments of *Acacia/Vachellia* (probably *V*. *tortilis* (Forssk.) Galasso & Banfi subsp. *raddiana* (Savi) Kyal. & Boatwr.), sometimes also *Tamarix* sp. (probably *T*. *aphylla* (L.) H.Karst.), more rarely *Nerium oleander* L., *Cupressus* sp. (probably *C*. *dupreziana* A.Camus) or *Ficus* sp.
- *Barks*: fragments of rhytidome, or rind, of stems or branches of woody plants. They are mostly not identifiable, but they are probably attributable to *Acacia/Vachellia* or *Tamarix*, being the most frequent woody species in this area; only one fragment resembled *Phoenix dactylifera* L.
- *Grass stems*: parts of caulis of Poaceae, clearly recognizable by their cylindrical hollow section and the structure articulated in nodes and internodes; some stems of Cyperaceae were recognized by the triangular section; in a few cases, the cylindrical stem, approximately 0.5–1 cm diameter, with epidermis longitudinally striated and a dense spongy pith, was attributable to species of Typhaceae family.
- *Leaves*: fragments of mummified leaves (rarely entire leaves); some of them were desiccated leaves of Poaceae, recognizable for their typical narrow, elongated shape with apex acute and parallel veins.
- *Wood Sticks*: splinters, sticks or small pieces of wood whose shape and section, usually flattened and irregular, suggests they are the product of a break (not necessarily intentional).
- *Roots*: fragments of root systems, usually easily recognizable by their twisted and irregular morphology and the presence of lateral rootlets. They were parts of the root system of Poaceae, recognizable by their twisted shape and small diameter (1.5–2 mm maximum in the thickest roots).
- *Twigs*: fragments of branching of woody plants, easily distinguishable by their circular or sub-circular cross-section; rarely identifiable at the species level for their too small diameter, in a few cases they were little branches of *Acacia/Vachellia*.
- *Thorns*: sharp elements with enlarged base and pointy apex, made of sclerified tissues, belonging to epidermal tissues of woody plants; some of them may have been lost from the barks of trunks or branches of the spiny acacia trees.
- *Inflorescences*: groups of flowers or spikelets of Poaceae (Paniceae, Andropogoneae), borne by the same stem, which is often divided into several branches; in few cases, the inflorescences were identified as *Pennisetum* sp.
- *Fruits*: fruits of angiosperms, nearly always fleshy (drupes). Fruits of *Balanites aegyptiaca* (L.) Delile were common, largely being well preserved endocarps, sometimes still maintaining the fruit flesh; fragmented pods of Fabaceae (possibly *Cassia*, in a few cases *Acacia/Vachellia*), some drupes resembling those of *Celtis* and a few syconia from *Ficus* were also observed. A single case of gymnosperm, included in this category for simplicity, is the strobilus of *Cupressus dupreziana* A.Camus. Very few fruits of *Phoenix dactylifera* L. with preserved pericarp and esocarp were also found.
- *Seeds*: seeds from Sieve and Volumetric sampling belonging to different angiosperm species; among them, *Acacia/Vachellia*, and *Citrullus* (*C*. *colocynthis* (L.) Schrad.), and also fragments of *P*. *dactylifera*.

### Artifacts

**Fig 4:**
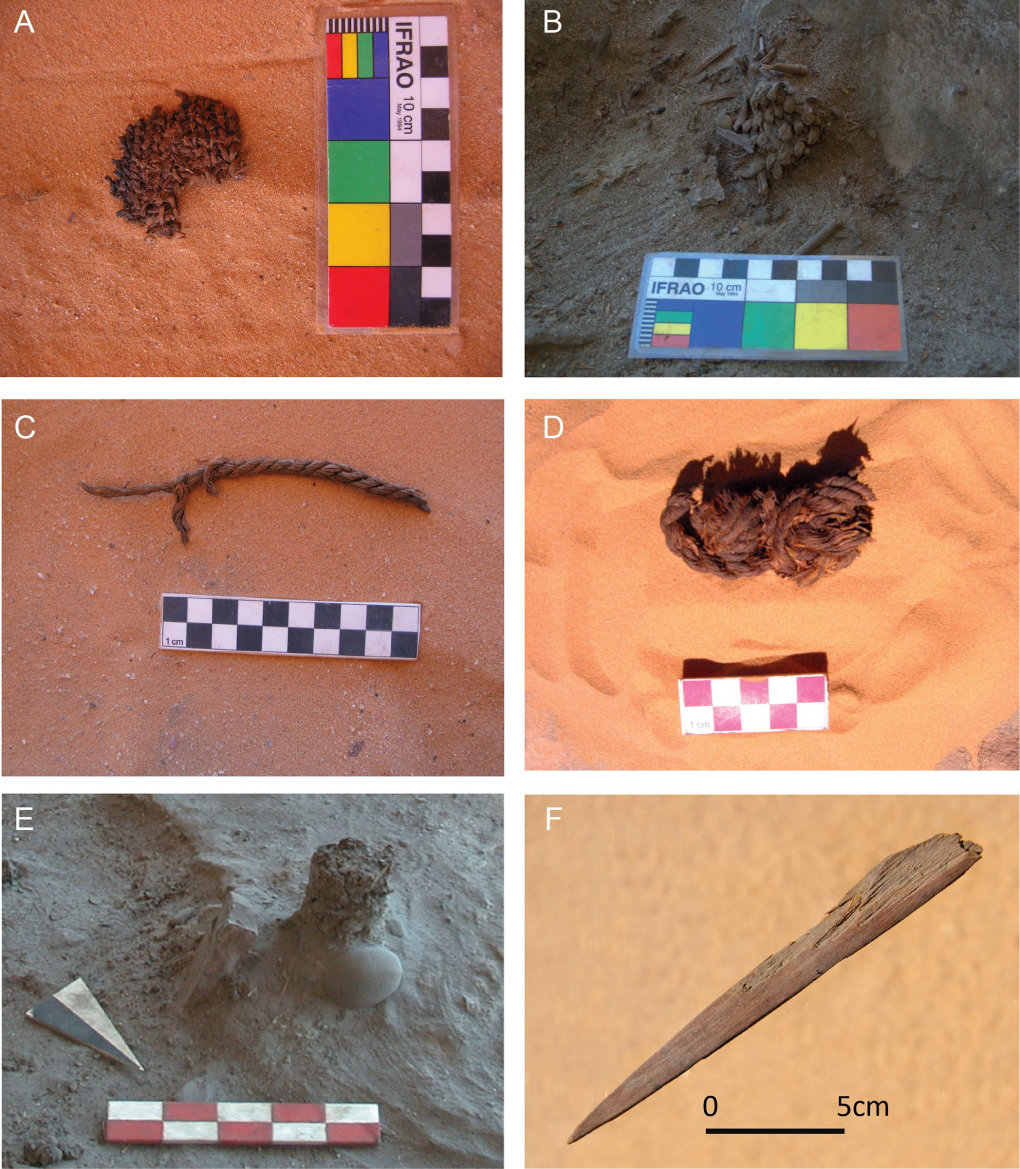
Some examples of artifacts. A) and B) basketry remains (LA3); C) (MP1) and D) LA3) cordage; E) wooden pole (LP1); F) a wooden point (LA3) (photos ©The Archaeological Mission in the Sahara).

This class includes four categories, totaling 121 elements, often in an exceptional state of preservation (Fig 4). Their low numerical incidence does not allow for a statistically relevant spatial examination, but they have high functional/interpretive value:

- *Basketry*: includes plant fiber artifacts, usually stems of herbaceous plants, twisted or interwoven into more or less long sections, thus defining a recognizable weave (Fig 4A and 4B).
- *Cordage*: includes elements composed of filaments of heterogeneous materials (herb stems, bark and animal fibers), woven or twisted together (Fig 4C and 4D).
- *Wooden pole*: wooden structural element with a supporting function, well recognizable by the diameter of the rounded section and the development in the longitudinal direction (Fig 4E).
- *Wooden tool*: includes a range of worked wooden tools, clearly recognizable as such, of various shapes and sizes (e.g., hooks, points, and spatulas) (Fig 4F).

## Results: The spatial analysis

### Stratigraphic distribution and spatial patterning of the archaeobotanical record

Charcoals is the most widespread category (35% out of total), with close relationship with fire installations and fire related activities, a complex topic that will be subject of a forthcoming study.

The remaining most frequent classes of archaeobotanical remains are Barks (26% of total), Fruits (13% of total), Sticks (9%) and Twigs (10% of total) which together make up ca. 58% of the record (Table 4). Their ubiquity and frequency, combined with their inherent interpretative potential, make them the four categories under scrutiny here.

**Table 4:** Number of plant remains, by cultural phase.

Class	Category	Chronological sub-phases								Total
		LA1	LA2	LA3	EP1	EP2	MP1	MP2	LP1	
**Ecofacts**	Charcoals	190	988	1921	1578	664	171	1325	320	**7157**
	*Barks*	323	1565	1131	525	675	117	886	109	**5331**
	Culms of grasses	50	292	319	231	155	21	211	20	**1299**
	Leaves	0	1	3	0	0	0	3	0	**7**
	*Fruits*	19	178	464	414	482	69	888	118	**2632**
	Inflorescences	1	5	1	2	0	0	2	0	**11**
	Wood *Sticks*	79	587	524	223	172	35	211	23	**1854**
	Roots	1	3	9	7	11	1	27	2	**61**
	*Twigs*	117	503	481	305	210	43	314	48	**2021**
	Seeds	0	0	1	3	8	2	8	0	**22**
	Thorns	1	18	0	2	0	0	2	1	**24**
** Artifacts **	Cordage	1	6	10	5	5	10	1	0	**38**
	Basketry	4	14	25	5	0	4	1	0	**53**
	Wooden artifacts	1	3	10	4	1	0	6	0	**25**
	Wooden pole	1	1	0	1	1	0	0	1	**5**
***TOTAL***		***788***	***4164***	***4899***	***3305***	***2384***	***473***	***3885***	***642***	***20540***

The high rate of frequentation of the site, though with chronological trends and differences, is directly related to the abundance of plant remains. As expected, much of plant remains comes from Matrices, i.e., sands with abundant organic materials, regardless of their socio-cultural context, followed by Fixtures while the other ACs host less records (Table 5).

**Table 5:** Percentage of plant remains, by Archaeological Contexts (ACs).

Class	Category*	Archaeological Context			
		Matrices	Fixtures	Burials	Physiogenetic
**Ecofacts**	Charcoals	71.1	28.8	0	0.1
	*Barks*	81.5	17.7	0	0.7
	Culms of grasses	82.2	17.3	0	0.5
	Leaves	100.0	0	0	0
	*Fruits*	82.3	17.7	0	0
	Inflorescences	54.6	45.4	0	0
	*Sticks*	78.6	21.0	0.1	0.3
	Roots	93.4	6.6	0	0
	*Twigs*	83.5	15.4	0	1.1
	Seeds	81.8	18.2	0	0
	Thorns	91.7	8.3	0	0
**Artifacts**	Cordage	64.5	35.5	0	0
	Basketry	74.3	22.3	2.8	0
	Wooden tool	88.0	12.0	0	0
	Wooden pole	66.7	33.3	0	0

*in italics the four categories considered for geostatistical and spatial analysis in this study.

Matrices represent the most common type of Archaeological Context found in the site and may function as good sedimentary traps where organic remains are retained after the depositional event. Interesting is also the association of ecofacts with Fixtures, which by representing “*structures évidentes*” [54], may be related to activities directly linked to their use or functional domain [21, 55], either as *de facto*, primary or secondary refuse [56, 57]. Functional interpretation of these distribution depends on contextual analysis and variables, for which a systematic description is needed and accomplished through the interpolation of the spatial analysis results and an *ad hoc* interpretative framework [15, 16, 42, 58, 59].

### Chrono-cultural spatial patterns of selected archaeobotanical remains

**Fig 5:**
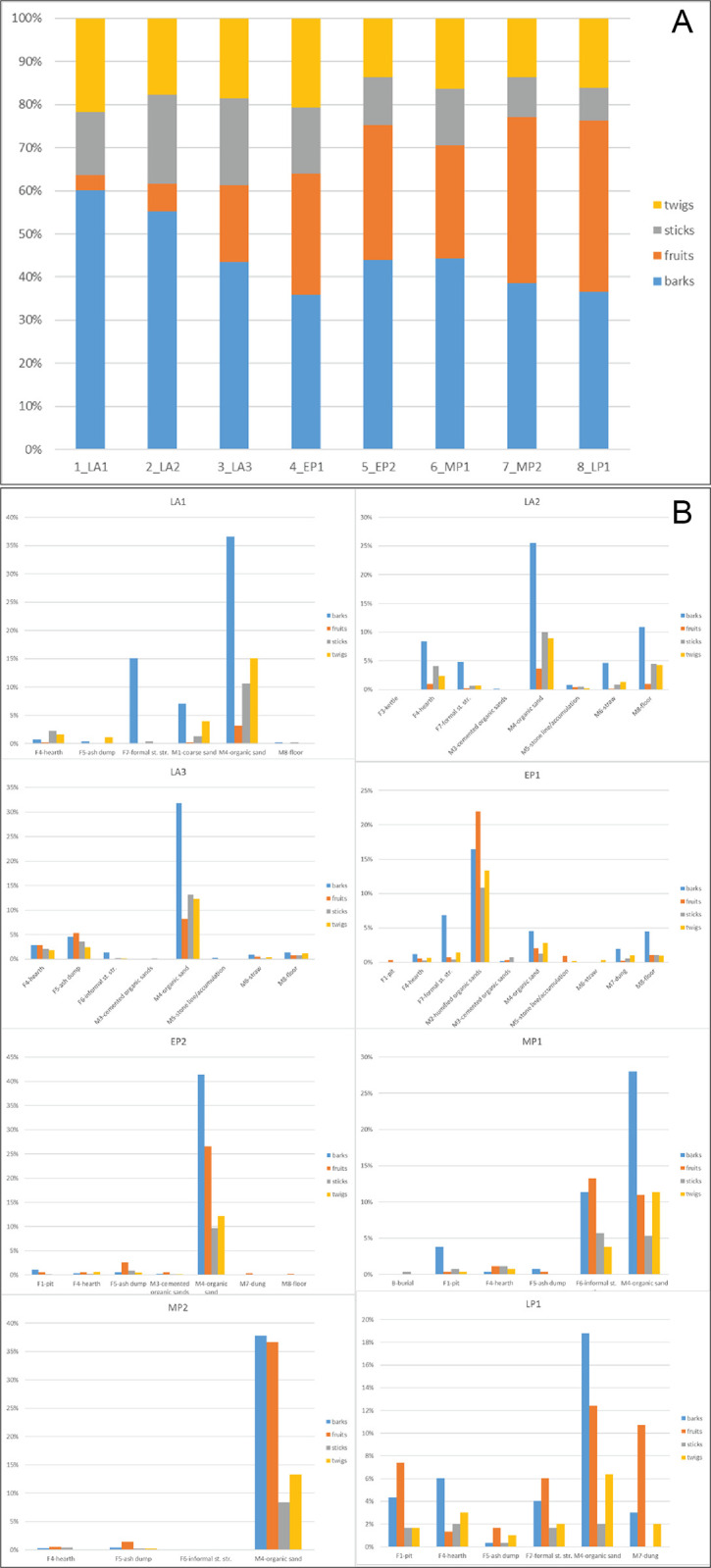
Percentage and trends of selected ecofacts. A) Selected ecofacts (Barks, Fruits, Sticks and Twigs) by sub-phases and B) divided by main ACs.

The bulk of archaeobotanical remains (Barks, Fruits, Sticks and Twigs) predominantly originate from the Late Acacus phase (Fig 5). This is attributed to the nature of occupation, which has been interpreted as semi-residential and characterized by prolonged periods of occupation [21]. A steady decrease in total raw frequency of plant remains is the trend from the Early Pastoral onwards among all categories except for Fruits, which show an inverse trend. Indeed, a second peak in total frequency is recorded in the Middle Pastoral 2 sub-phase, driven mostly by the great number of Fruits which duplicate in comparison to all the preceding sub-phases.

The material remains of the Late Acacus 1 sub-phase (LA1) were brough to light only in the Northern Sector. Among the categories under study (S1 Table), Barks and Twigs are the most represented, followed by Sticks and Fruits. Most of the material comes from matrix layers and to a lesser extent from some fixtures, 15% of the Sticks from hearths (F4) and 25% of the Barks from formal structures (F7).

The archaeological deposit of the Late Acacus 2 (LA2) is present in all the excavated sectors, as mirrored by the large quantity of plant remains (> 2800 items). Barks and Sticks were the most represented, largely coming from matrix-type layers, especially organic sands (M4) and floors (M8).

Late Acacus 3 (LA3) yielded also a high amount of material (2600). Barks is the most common, while Sticks, Twigs and Fruits show similar amounts. Most of the ecofacts (an average of 66%) come from organic sands (M4), followed by ash accumulations (F5) (S1 Table).

Less than two thousand ecofacts comes from the Early Pastoral 1 (EP1) archaeological contexts, mainly organic sands (M2 and M4) and to a lesser extent from some fixtures, such as formal stone structures (F7) (S1 Table).

About 1400 remains come from the Early Pastoral 2 (EP2), with frequencies fairly similar to the previous EP1, both quantitatively and stratigraphically: however, Fruits are more common. The M4 organic sand represents the major depositional context (Table 5).

266 plant remains come from the Middle Pastoral 1 (MP1) sub-phase, an exiguity largely due to post-depositional phenomena. Most of them come from organic sands (M4), followed by some structural elements, particularly informal structures (F6) (S1 Table).

The Middle Pastoral 2 (MP2) accounts for about 2200 plant remains, with a particular high frequency of Fruits and Barks. The stratigraphic context of MP2 is made almost exclusively of organic sand layers, which together with fire-installation, i.e., hearths, are the main features of the occupation layers (S1 Table).

Slightly less than 300 remains come from the Late Pastoral 1 (LP1), with Barks and Fruits more numerous. The stratigraphic context is mostly represented by organic sands (M4), although structural elements are also common (S1 Table).

The deviation from Complete Spatial Randomness CSR observed in the Quadrat Counts Test QCT analysis for the spatial distribution of botanical remains could potentially be attributed to various biases introduced during the excavation process. However, the consistent spatial behavior differing from CSR and the presence of different directionality, as observed through both QCT and SPP (Spatial Point Pattern), suggest that there may be causal factors at play, including primary and second-order effects.

Specifically, for the Late Acacus horizons, a strong deviation from CSR is observed for Barks, Sticks, and Twigs (S2 Table). This indicates a non-random distribution and clustering of these materials within the sub-phases. On the other hand, in the Middle Pastoral layers, there is a greater susceptibility to deviations from CSR for Fruits (S1 Fig; S2 Table).

These findings suggest that there may be underlying factors influencing the distribution of materials in the archaeological context being studied and the nature and causes of it are further addressed by a contextual approach.

## Organization and use of the space

### Activities and functions: An interpretative framework

The plant materials here observed could have been used in different ways and differently entered the archaeological record, hence indicating different types of activities and use of spaces. Nevertheless, based on the archaeobotanical and ethnobotanical research carried out as part of archaeological research at Holocene sites in the region and other chronologically comparable contexts [60–66], some broad functional categories can be hypothesized (Table 6). Possible plant uses can be identified in what concerns the realm of dwelling structure and habitation and living space, from their construction to maintenance and dismission. Wood, together with leaves, is used as the main structural element in regional vernacular architecture, as documented by ethnographic and ethnobotanical research in North Africa (e.g., among the ethnic groups Tebu in the Southern Sahara and Tuareg in the Central Sahara; [67–70]).

**Table 6:** Selected categories of plant remains and their possible uses, based on ethnobotanical and ethnographic research.

Ecofact categories	Possible/Attested use
Barks	building material
	bracing elements
	floor installation
	tannin extraction
	phytomedicine
	paneling
	roofing
	fuel
Fruits/seeds/flowers	food
	fodder
	medicinal use
	oil extraction
Wooden Sticks	building material
	bracing elements
	fuel
Twigs	building material
	bracing elements
	roofing huts
	bedding
	food preparation (e.g., stirring etc.)
	food enhancement (e.g., tamarix salt)

*Barks* and *Wood sticks* can be associated as structural elements with functions of support or reinforcement. Bark roofing, paneling and floor installments are recorded quite diffusely in vernacular architecture both archaeologically [71–73], and in ethnobotanical accounts. Moreover, barks can be processed to extract tannins, saponins, lipids and antimalarian compounds [74, 75].

*Cordage*, when associated to Barks, Twigs, or Wood sticks, can be indicative of its use as binding device for erecting hut walls, panels, windbreaks or enclosures [32, 76].

*Twigs* or branches can be associated with uses in the domestic sphere, such as bedding, processing waste and fuel, or roof coverings for huts [3, 62, 65]. This use as building material is common among the Tuareg kel Tadrart: wood branches of tamarisk species are used for the walls and internal divisions, whereas acacia branches are employed for the hut cover [77]. Also, plant bedding is widely documented in ethnographic accounts whereas more difficult and painstaking is its archaeological preservation and identification [78].

*Leaf* arrangements, matting, and similar devices have been identified in hunter-gatherer archaeological sites through micromorphological approaches, though occurrences of unambiguous macro-remains are rare [62, 78–80].

In the Sahara, present-day desert populations such as the Tuareg Kel Tadrart still use these categories of ecofacts [77], and adopt the ‘multipurpose approach’ on exploiting the same plant species for food, medicine and other uses [65]. Pollen accumulations as well as the technological analysis of basketry remains also testify to the skilled transport of inflorescences and grass stems at Takarkori [25, 32]. An exceptionally impressive find is the documented use of a bundle of grass stems, partially burnt, in connection with a fireplace (Fig 3B).

*Fruits* are among the most common archaeobotanical remains that are prevalently interpreted as food evidence, as accumulation, storage or votive offer depending on the specific context. As seen above, the record of Takarkori includes many drupes of *Balanites aegyptiaca*. This tree species, typical of the arid zones of the Sahara and the Sahel, is used today by the Tuareg Kel Tadrart for food, as well as fodder for camels and goats: in the dry season, animals can also consume its leaves and branches [66]. In many parts of Africa, the Middle East and the Indian subcontinent, this is a known oil plant and different plant organs, from fruits to roots, are used for the treatment of some ailments, whose efficacy is corroborated by phytochemical research [81]. Fruits, seeds and charcoals of the desert date have been recorded in the Tadrart Acacus [79], and are also reported from Egyptian predynastic settlements and cemeteries [e.g. 82, 83 and references therein]. Remarkable is also the presence of seeds of *Citrullus colocynthis*, a viny plant spread even today on desert sands. The plant has known purgative effects, and it is a vermifuge. The dried pulp of the peeled fruit without seeds is used also for other medicinal purposes. The powder is irritating to mucous membranes and is usually administered mixed with other drugs on account of its griping action [84].

Within these frames of references and analogies (Table 6), and while still considering cautionary tales, an interpretative proposition regarding the arrangement of space and activities within the site is here proposed.

### Space and activities during the Late Acacus

#### Late Acacus 1 (10,200–9400 years cal BP)

**Fig 6:**
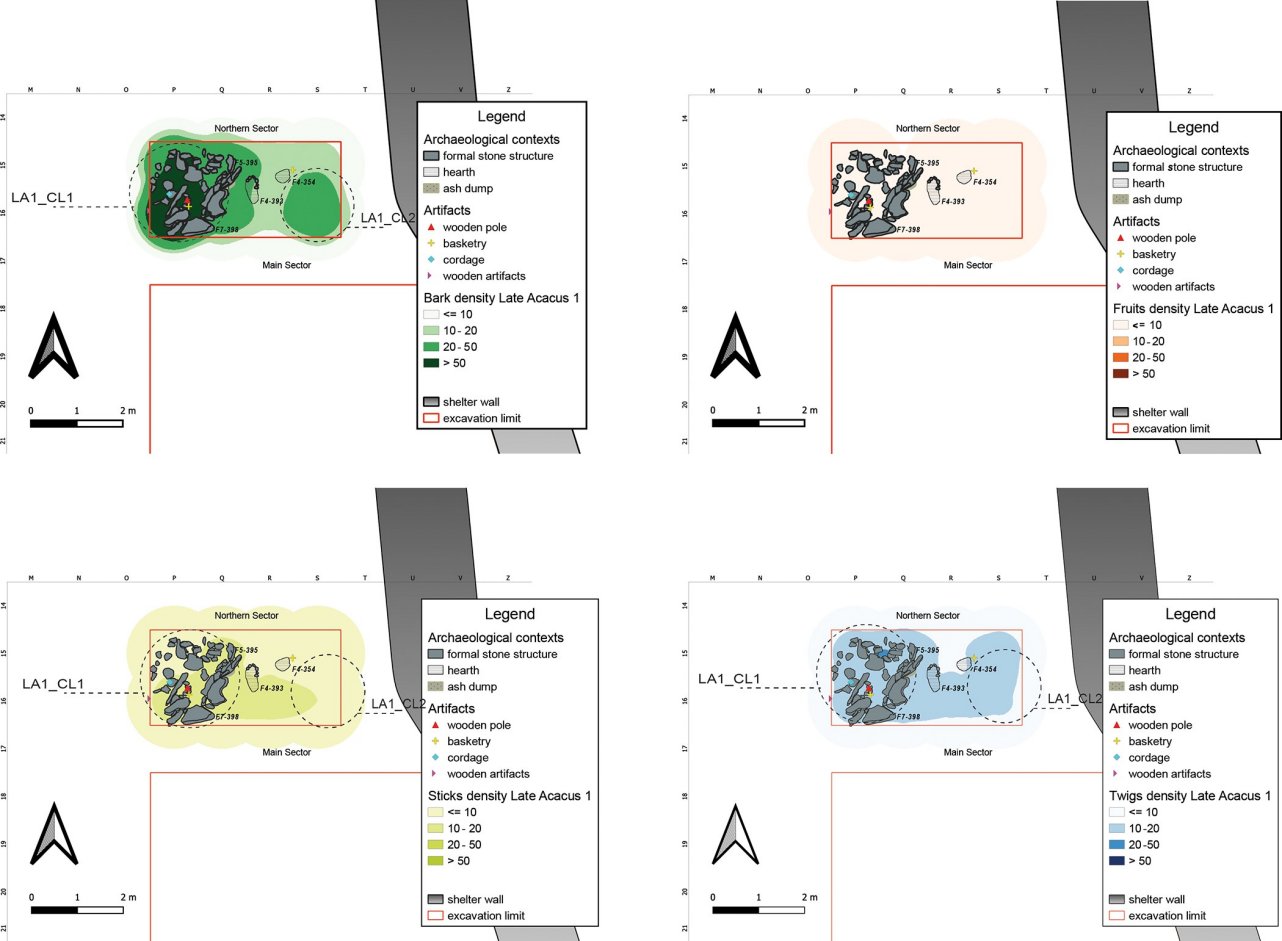
LA1 Kernel Density Estimation. LA1 Kernel Density Estimation of selected ecofact categories (Barks, Fruits, Sticks and Twigs), with plotting of relevant artifacts discussed in the text.

The LA1 horizon, relating to the first occupation of the site by hunter-gatherer-fisher groups, was intercepted exclusively in the Northern Sector (Fig 6). This area is characterized by the presence of important structural elements such as a formal stone structure composed of large slabs, likely associated with two hearths on the east towards the shelter wall. The interpretation of the structure as a hut is supported by the high number of Barks some of large size, which together with the presence of an actual wooden post are interpreted as the remains of one or more support posts LA1_CL1, (Table 7).

**Table 7:** Spatial clusters by chronological sub-phases.

Chronological sub-phase	Cluster ID	Cluster location and description	Main ACs and ID	Ecofacts+ artifacts	Spatio-functional interpretation
Late Acacus 1	**LA1_CL1**	NS. High number of *Barks* (81 fragments, ca. 25% out of a total of 323)	F7 –formal stone structure (398)	*Barks*, *Twigs*, Wooden post	Hut with plant roofing sustained by wooden posts
	**LA1_CL2**	NS. Fragmented *Fruits* strictly outside of the structure, between the hearths and the shelter wall	F4—hearth (393, 354)	*Fruits*	Processing area of food resources
Late Acacus 2	**LA2_CL1**	NS. High frequency of *Barks* and *Sticks*. Their distribution follows the circular outline of the stone arrangement	F7—formal stone structure (374)	*Barks* (329 items, ca. 20% out of a total of 1565) *Sticks* (82 items, ca. 14% out of a total of 587). Wooden pole	Hut with perishable wall and roofing
	**LA2_ CL2**	NS. High presence of *Fruits* and artifacts, especially basketry and cordage	F4-hearth (378) M4-organic sand (376, 372, 379, 383) M8-floor (384, 387)	*Fruits* (37 items, ca. 20% out of a total of 178) Basketry	Food processing area; Outside activity area with rug or mat lined floors
	**LA2_CL3**	Located in the MS, it associates with formal stone structures	F7—formal stone structure (174); F1—pit (filling) (220, 228), F3 (229, 240, 242)	*Fruits*	Storage and processing area
	**LA2_CL4**	Located in the west-central area of MS, perimetral to F7	F7—formal stone structure (180)	*Barks* and *Sticks*	Wooden fence with basal stone inlay
Late Acacus 3	**LA3_CL1**	MS. Several artifacts found in a defined zone between the shelter wall and the other clusters	F5-ash dump (163) F6-informal stone structure (151) M8-floor (107)	Basketry (13) Cordage (12) Wooden tools (9)	Multifunctional area
	**LA3_CL2**	MS. Located south of the main structure F7-131. *Barks* are more frequent	F7—formal stone structure (131)	*Barks* *Sticks* *Twigs*	Roofing Walling Hut
	**LA3_CL3**	Center of MS	F4 –hearth (71, 124, 197, 152) F5-ash dump (249) F6—informal stone structure (261, 151) M4-organic sand (103,69, 132) M8-floor (107)	*Barks* *Sticks* *Twigs*	Roofing Walling *Structure latent*
	**LA3_CL4**	Located in the MS southern part. Characterized by fire-related structures and ash accumulations with many *Fruits*	F1- pit (filling) (99) F4-hearth (97, 134) F5-ash dump (145, 138, 121, 168, 287, 267, 163) F6- informal stone structure (153, 154)	*Fruits* 139 remains, ca 30% out of a total of 464	Discard/for secondary refuse management
Early Pastoral 1	**EP1_CL1**	MS shelter wall	F7—formal stone structure (68–67) F4 –hearth (88–92) F1 - (91)	*Barks* Basketry Cordage Wooden tool	Informal dwelling area
	**EP1_CL2**	MS southwestern part. Associated with structural/formal evidence, and a wooden post. *Barks*, *Twigs*, and *Sticks* show similar accumulation densities. *Fruits* form a well-defined cluster	F1 –pit (filling) (85–86) F4—hearth (81) F5—ash dump (80)	*Barks* *Twigs* *Sticks* Wooden post *Fruits* (76 ecofacts -14% of the total).	Processing area Food/Resource storage area (?)
	**EP1_CL3**	Southern part of MS. Overlapping clusters of *Barks*, *Sticks*, and *Twigs*. *Fruits* show a richer and denser cluster	F1-pit (269) M2 –humified organic sand (38)	*Barks* *Sticks* *Twigs* *Fruits* (102 items -25% of the total).	Processing Storage Maintenance
	**EP1_CL4**	Near the shelter wall in MS in proximity of burial H6	B- burial (H6) (proximity)	*Barks* *Twigs* *Sticks*	Burial coating Inhumation wrapping
Early Pastoral 2	**EP2_CL1**	MS northeastern part.	F4—hearth (73)	*Barks* Wooden post *Sticks*	Wooden structure (hut/windbreak)
	**EP2_CL2**	Location in the MS inner corner. Cluster with high density of fruits in low state of preservation associated with concentrations of *Sticks* and *Twigs*.	F1—pit (filling) (62, 64, 87, 244) F4 –hearth (61, 63) F5 –ash dump (59, 57, 60–270).	*Fruits* (118 items; 8% of the total) *Sticks* *Twigs*	Secondary refuse management
Middle Pastoral 1	**MP1_CL1**	NS central-eastern area.	F6—informal stone structure (360–361)	*Barks*, *Fruits* Basketry, *Sticks* *Twigs*	Temporary shelter
	**MP1_CL2**	MS southeastern part. Clusters of varying intensities of different remains.	F6—informal stone structure (246)	*Fruits* (51%) *Barks* *Sticks* *Twigs*	Secondary refuse management
Middle Pastoral 2	**MP2_CL1**	MS southern area	F4 –hearth (233, 231, 209, 198, 204, 206, 194) F5 –ash dump (207, 243, 227),	*Barks* *Twigs* Wooden tools Basketry	Small animal enclosure
	**MP2_CL2**	MS southern area	F6—informal stone structure (33)	*Fruits* *Sticks*	Animal management /Fodder
Late Pastoral 1	**LP1_CL1**	MS northeastern part. Gradually decreasing towards south. Hotspot in association with *fixtures*.	F7 –formal stone structure (21), F1 –pit (filling) (29, 23, 19, 16), F2—post hole (filling) (18) F4 –(24, 26, 8, 5, 4)	*Barks* *Sticks* *Twigs*	Multifunctional area. Food processing outside dwelling
	**LP1_CL2**	MS central area. towards the shelter wall.	M7- dung (6) F2- post hole (filling) (18)	*Barks* *Sticks* *Twigs* *Fruits* Wooden pole	Fenced area for animal management// fodder

Dwelling structures realized with the use of light wooden structures in various arrangements have been hypothesized at some archaeological sites, suggesting the employment of poles made of branches or tree roots (often of *Acacia/Vachellia*, or *Tamarix* sp.) [85]. The presence and distribution of Twigs around the structure may be related to the remains of some kind of installation with covering function, hypothesizing a composite framework made of trees and shrubs. The clustering of this material category only in this part of the quadrants may moreover corroborate such reconstruction, just in perpendicular location where the vault/roof would have been located.

All Fruits are strictly outside of the structure, in the area between the hearths and the shelter wall, and many of them have a fragmentary state of conservation. Albeit with low numbers, they could define a processing area of food resources, LA1_CL2. The negative correlations between Fruits and the other ecofacts (Barks, Sticks and Twigs; S3 Table) would confirm that the fruits were concentrated in a specific area of food processing.

#### Late Acacus 2 (9500–8600 years cal BP)

**Fig 7:**
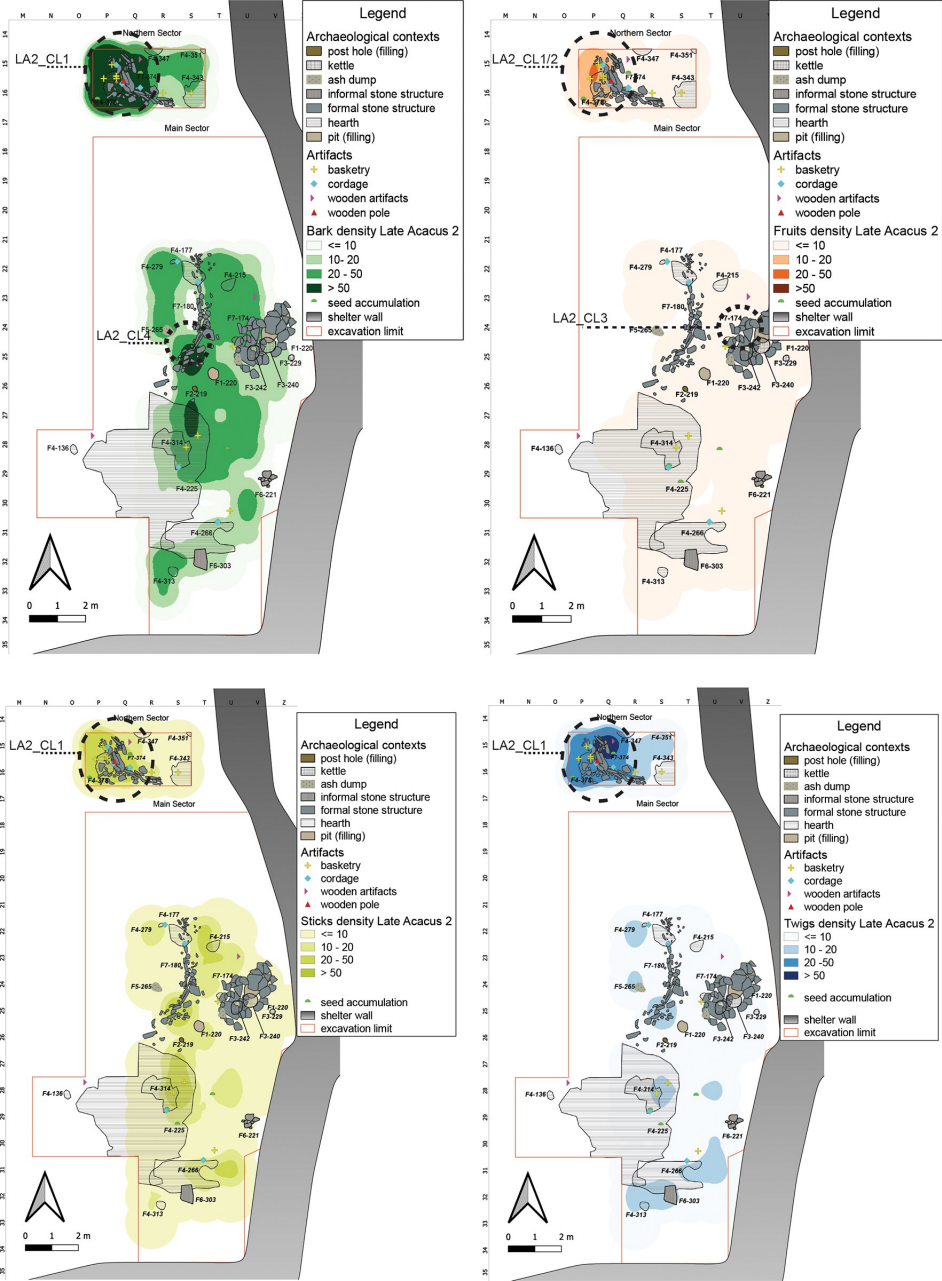
LA2 Kernel Density Estimation. Kernel Density Estimation of selected ecofacts (barks, fruits, sticks and twigs) in LA2, with plotting of relevant artifacts discussed in the text.

**Fig 8:**
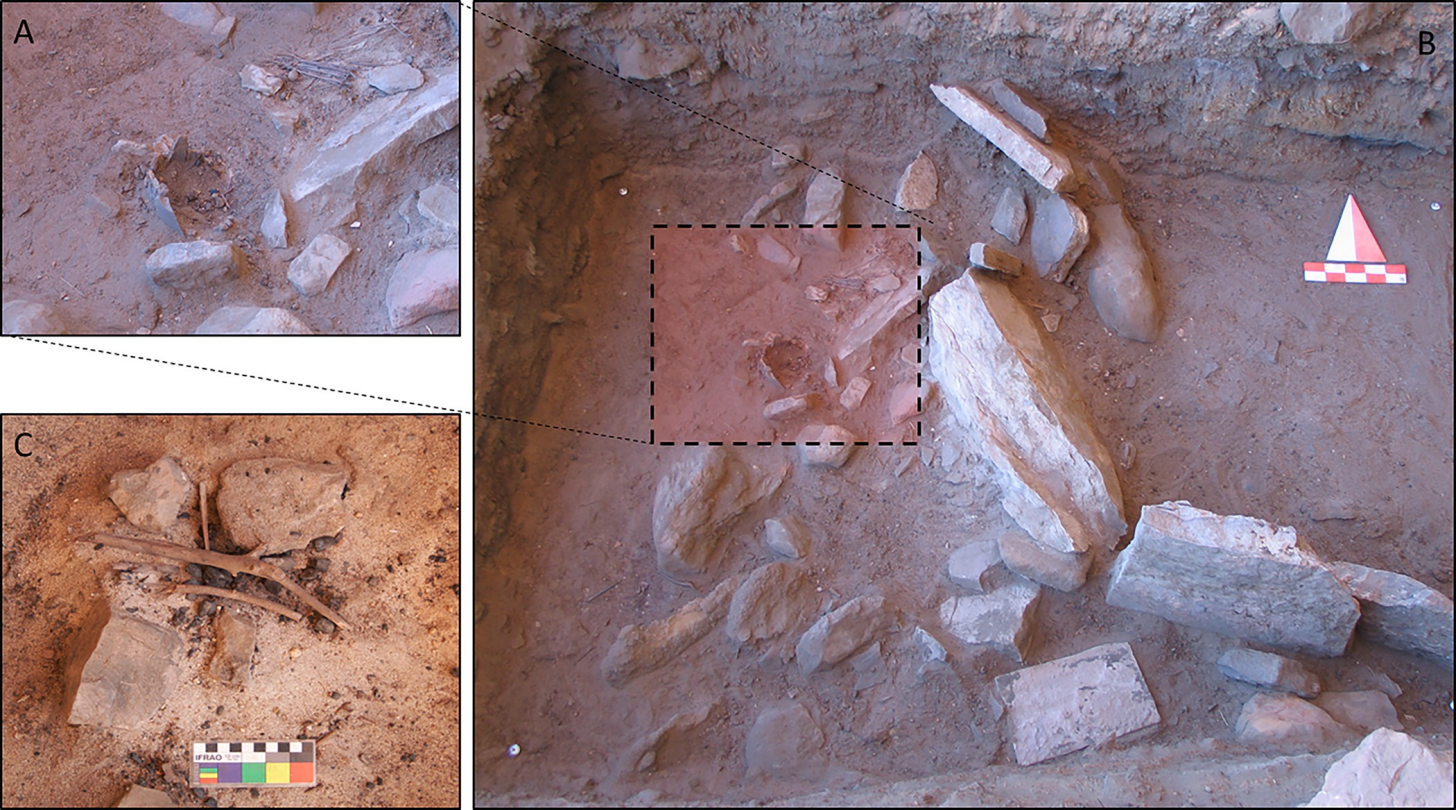
Structural and archaeobotanical features from LA2 horizon. A) close up of the wooden pole (bottom) and twigs associated with the B) hut-like structure F7-374; and C) a fireplace with still in place preserved twigs and sticks.

The prolonged, semi-residential occupation, as well as advanced forms of resource management of LA2 horizon are reflected in the frequency and distribution of the archaeobotanical record under study (Fig 7). The analyses allowed the identification of an area of high density of remains in the Northern Sector (**LA2_CL1**) in association with a structure interpreted as a hut/dwelling structure. The frequency of Barks and Sticks suggest their use as building material (the perishable walls?), or as roofing supported by the remaining pole (Fig 8). Their distribution follows the almost circular outline of the stone arrangement, indicating its spatial and probable structural association. Pearson’s r coefficient confirms the Barks-Sticks positive association with a particularly high value (S3 Table), supporting an intended, joint use of these ecofacts, presumably as part of the same wood materials.

In this same area (**LA2_CL2**), the presence of Fruits and various artifacts, especially basketry and cordage, may define an area for processing activities. Millet (*Panicum* sp., including *P*. *laetum* Kunth) and sorghum (*Sorghum* sp., namely *S*. *bicolor* (L.) Moench), for instance, were systematically harvested, cultivated and stored inside baskets [4, 32], or in shallow concavities dug into the sandstone bedrock, the so-called kettles [86]. The ethnobotanical and technological study on basketry and cordage clarified the type of use of these artifacts as remains of containers made of woven plant fibers, used for the storage of wild cereals [32]. However, given their spread presence across the site, the interpretation of these elements as parts of woven mats, probably connected to dwelling areas and implicated in the preparation of bedding or floor systematization/levelling through matting and rug-like furnishings cannot be ruled out [62, 78]. In the Main Sector, a space with possible storage and processing purposes was evidenced by well-preserved fruits and can be identified in the central area close the shelter wall, **LA2_CL3**, where also formal stone structures, pits, and kettles are found.

The central-southern zone of the Main Sector is bordered to the west and south by the presence of a rather extensive fire-related area likely connected to the main domestic-dwelling unit, characterized by high maintenance given the dearth of remains. In the central-western area, the formal stone structure (F7–180) was interpreted as an area for Barbary sheep (*Ammotragus lervia*) corralling, based on the abundance of fodder and animal coprolites [87]. This interpretation is now further supported by the abundance of Barks and Sticks around the perimeter of the structure, and **LA2_CL4** might represent ephemeral remains of the perishable parts of a wooden fence reinforced with stones at the base. Examples of such structures can be found in some of the ethnographic contexts investigated by [76], as part of their study of the continuity between prehistoric and historic animal management practices. The spatial visual correspondence between Barks and Twigs is supported by high r-value (S3 Table), indicating a positive correlation between these materials. A good correlation was also found for the association Barks-Twigs and Sticks (S3 Table).

These agglomerations represent different functional zones within the archaeological site, each with specific features and potential purposes related to resource management, dwelling, processing activities, and animal management.

#### Late Acacus 3 (9000–8000 years cal BP)

**Fig 9:**
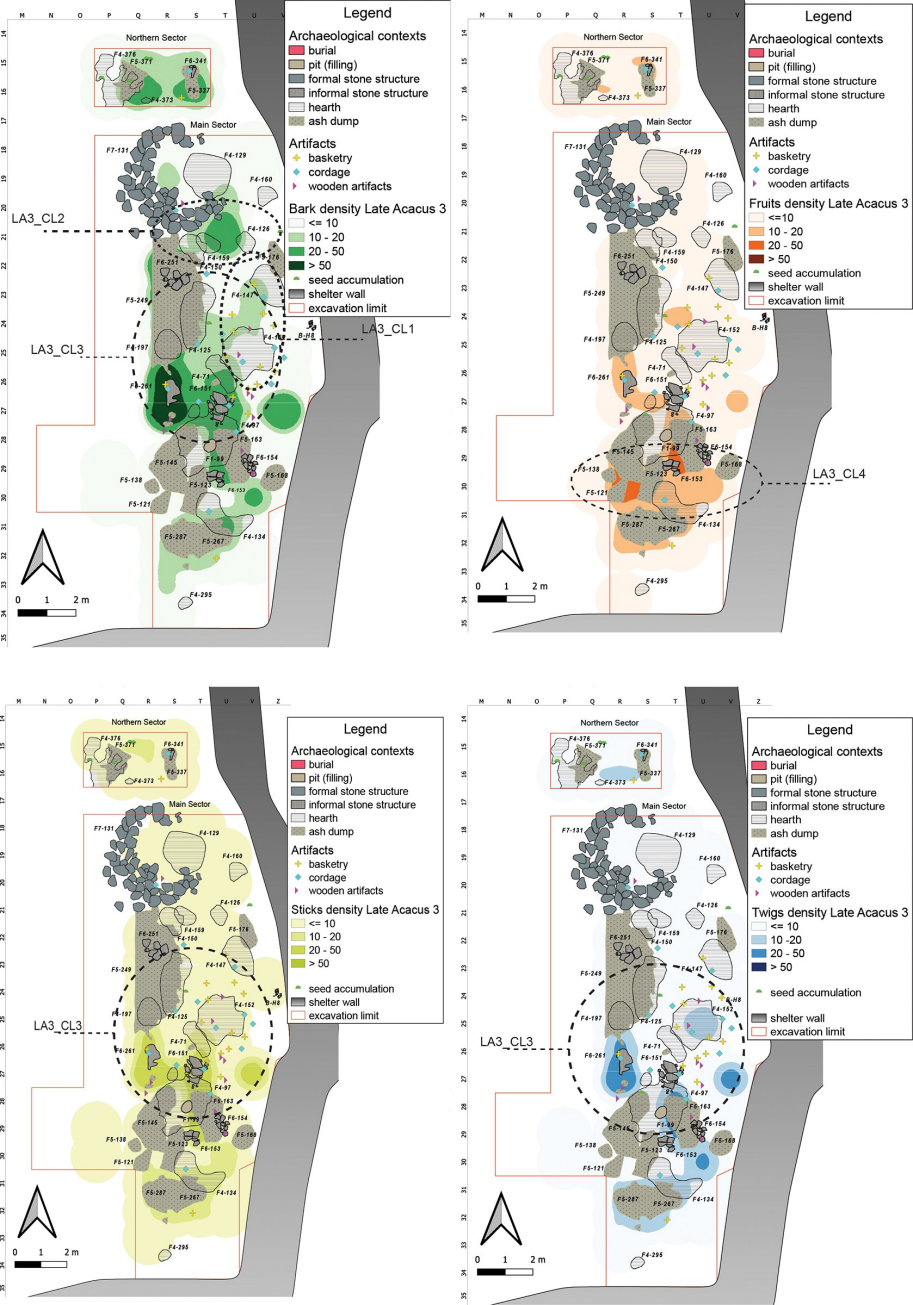
LA3 Kernel Density Estimation. Kernel Density Estimation of selected ecofacts (barks, fruits, sticks and twigs) in LA3, with plotting of relevant artifacts discussed in the text and indication of clusters specified in Table 7.

The LA3 occupation levels, characterized by several Fixtures, especially hearths and ash accumulation, are among the richest of plant remains with more than 80% from the central-southern and western parts of the Main Sector, leaving the northern and north-central space almost free. Several artifacts, **LA3_CL1**, found between the shelter wall and the clusters of ecofacts (Fig 9), are part of a large multifunctional area with external activities linked to a dwelling structure, a hut, 131, where **LA3_CL2** is localized.

The KDE highlights a clustering in the center of the Main Sector, **LA3_CL3**, where the spatial outline may hint to the presence of some kind of “*structures latentes*”, *sensu* Leroi-Gourhan. It is tempting to recognize in those characters the residues of a more formal structure resembling in fact hut-like features, disrupted and whose building materials could have been reused later. This same spatial layout is clearer for the actual hut structure 131, where we however observe different conservation dynamics and formation process.

Huts roofed by tamarisk branches and lined inside by mats have been hypothesized for other contemporary sites like the structure 1/90 at site E-75-6 in the Nabta Playa basin [88] and the already aforementioned Sudanese examples [85]. *Typha*- roofing for a hut like structure has been advanced for the remains unearthed at Uan Tabu [65, 89].

The stones arranged in a circular fashion of structure 131 may have functioned as structural reinforcement and bracing for the lightweight structure of wooden material of which the few remains in question here are left. The almost clean internal surface, void of macro-remains but for few small evidences, should be considered the outcome of cleaning activities as specified by various intra-site analyses [55, 90]. Bigger elements are usually thrown out whereas smaller ones are projected towards the liminal area, walls edges etc., or embedded in the earthen floor further compacted through successive trampling or “under the rug/mat” effects [78, 91–93] and testified also by the widely planar micromorphological structure of soils sampled in the site itself [25].

**LA3_CL4**, another area of interest in the southern part of the Main Sector, is characterized by numerous fire-related structures, especially ash accumulations which could be assigned to a discard maintenance area, for secondary refuse management (*sensu* [94]) as hinted by the fact that about 1/3 of the fruits in this area came from levels of ash accumulations and are either fragmentary or partially preserved.

These agglomerations represent various functional and structural aspects of the archaeological site, including dwelling areas, potential structures, and refuse management areas.

### Space and activities during the Early Pastoral

#### Early Pastoral 1 (8300–7600 years cal BP)

**Fig 10:**
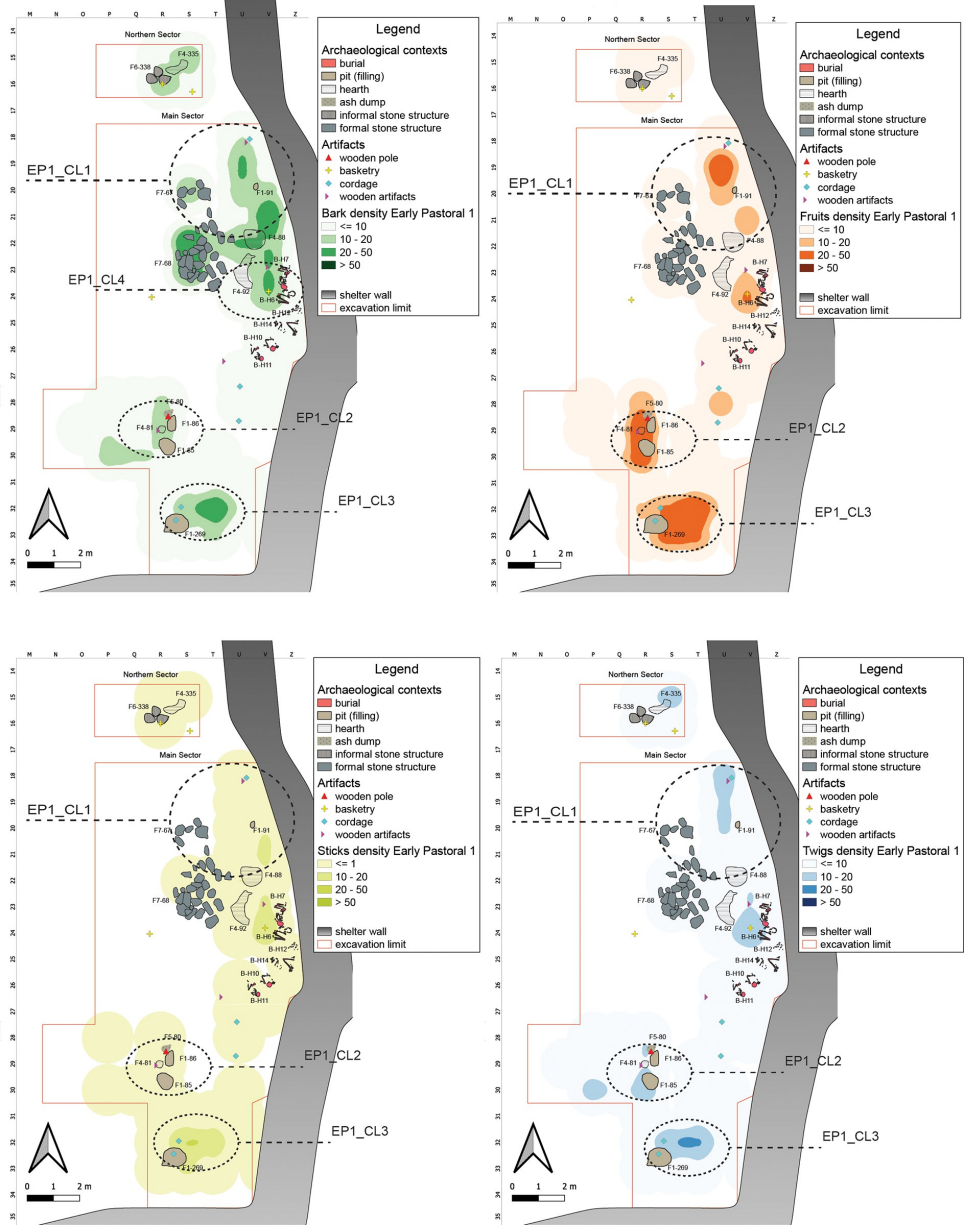
EP1 Kernel Density Estimation. Kernel Density Estimation of selected ecofacts (barks, fruits, sticks and twigs) in EP1, with plotting of relevant artifacts discussed in the text and indication of clusters specified in Table 7.

The earliest horizon of the Early Pastoral phase is characterized by important cultural changes [95]. The EP1 occupation shows stone structures and enclosure, as well as the presence of burials in close interaction with the habitation spaces [26]. The distribution pattern of plant remains in EP1 markedly differs from the previous sub-phases, with relatively very poor remains (Fig 10). Although post-depositional processes may in part explain this configuration–in particular the humification of organic matter–the presence of well-defined clusters associated with fixtures suggests specific uses of the shelter. In detail, only a low quantity of Barks and Twigs comes from the Northern Sector, whereas some major clusters are visible in the Main Sector.

In this context, the analysis of plant remains and artifacts define three main areas of interest, respectively, in the northeastern, southwestern, and southern parts of the Main Sector. The first cluster, **EP1_CL1**, is close to the shelter’s wall where several fixtures are located: a concentration of Barks in association with formal structures, likely remains of the perishable part of a dwelling structure, whereas the other plant categories are rather negligible or null in amount. Fruits cluster in the northeastern corner of this area. The presence of a few artifacts and structural elements such as two hearths and one pit could define an area related to the domestic sphere.

The second area of interest is in the southwestern part of the Main Sector, **EP1_CL2**, in association with the presence of some structural/formal evidence: two pits, one hearth, an ash accumulation, and a wooden post where Barks, Twigs and Sticks show similar accumulation densities. Here Fruits—only *Balanites aegyptiaca*—form a well-defined cluster. These desert dates show different state of preservation: some fruits still fleshy and other with only stones, some of them broken. This instance suggests food processing activities, probably to extract oil. Their association with pits could be related to the presence of some containers perhaps of perishable nature. At Takarkori, numerous grinding tools have been uncovered, yet no direct evidence link them to oil extraction [21, 96]. Combined molecular and isotopic techniques in pottery organic residue analysis revealed the presence of diagnostic plant lipids, including leaf waxes and seed oils, suggesting the processing of grasses, seeds and aquatic plants at the site [5].

The third area of interest is in the southern part of the Main Sector, **EP1_CL3**: Barks, Sticks, and Twigs form largely overlapping clusters, while Fruits again show a richer and denser cluster.

A marked cluster, shared by all the categories here considered, is in the vicinity of Burial H6, **EP1_CL4**.

**Fig 11:**
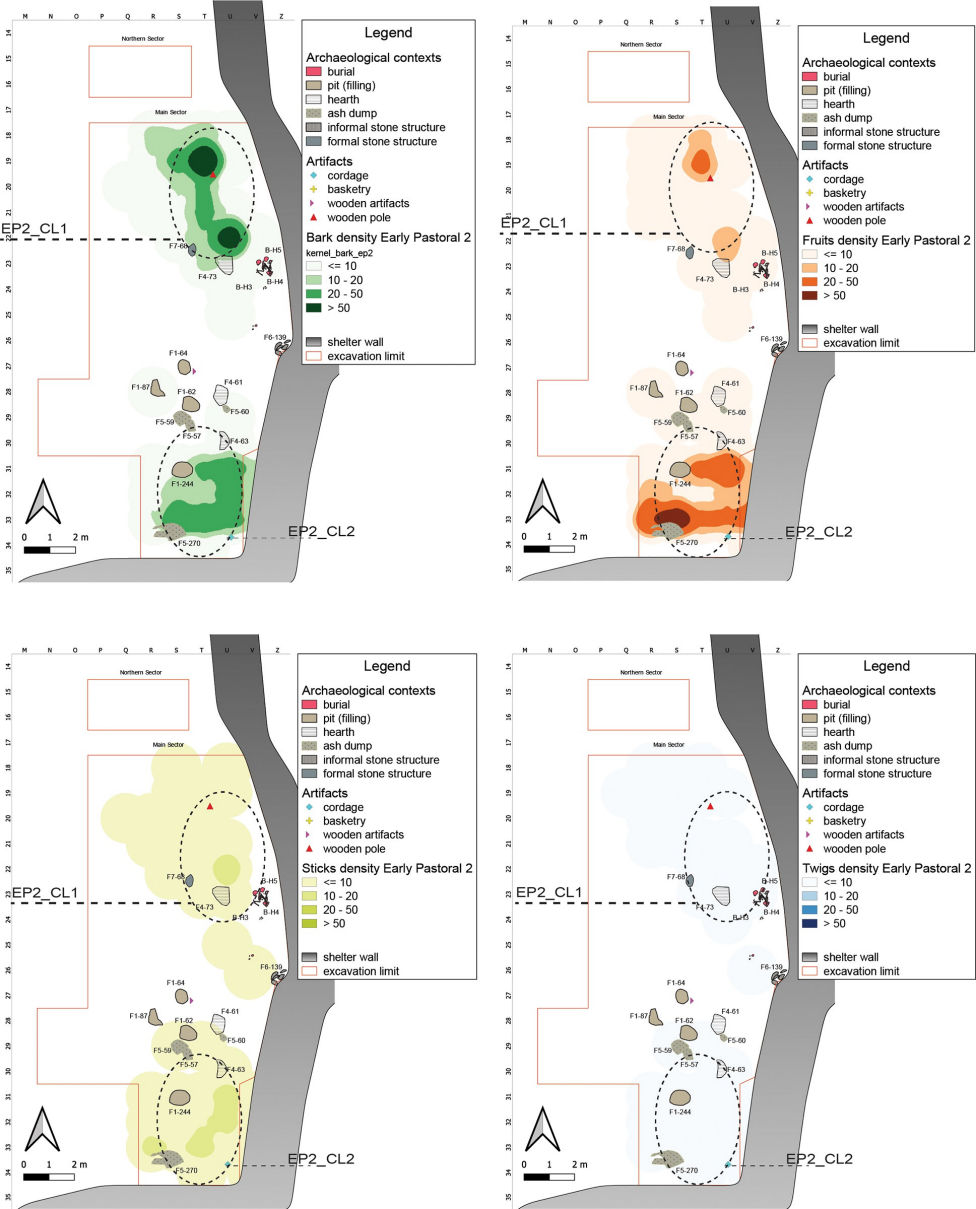
EP2 Kernel Density Estimation. Kernel Density Estimation of selected ecofacts (barks, fruits, sticks and twigs) in EP2, with plotting of relevant artifacts discussed in the text and indication of clusters specified in Table 7.

**Early Pastoral 2 (7800–7300 years cal BP).** The EP2 cultural horizon shows a degree of continuity with the previous sub-phase. Two main areas are defined, located in the Main Sector (Fig 11).

The first, **EP2_CL1,** in the northeastern part, features a cluster of Barks associated with a wooden post; together with a second cluster of Barks and a minor cluster of Sticks, a couple of meters south: these remains could represent the ephemeral evidence of wooden structure, either a hut or a windbreak. The presence of a hearth and minor concentrations of fruits support the interpretation of this area as a kind of dwelling context.

A second area, **EP2_CL2**, is of interest in the southern portion, between the shelter wall and fixtures such as pits ash accumulations, and hearths. The position in the inner corner of the shelter, together with the presence of fruits in low state of preservation partly associated with an ash dump as well as concentrations of sticks and twigs, could define a place for secondary refuse management.

### Space and activities during the Middle Pastoral

#### Middle Pastoral 1 (7100–6200 years cal BP)

The MP1 sub-phase is characterized by a more marked climate seasonality [97]. Compared with the Early Pastoral, we record major cultural discontinuities, expressed in particular by the exploitation of cattle secondary products and the full maturity of herding management [98]. These changes are reflected in the different setting of the shelter, characterized by the presence of small hearths, informal structures (F6), and burials (B). We record in this subphase an abrupt decrease in the number of plant remains (<2% out of the overall total) (S1 Table). Without again dismissing post-depositional erosional phenomena, this decrease can be also explained by the change in the shelter use, characterized now by a discontinuous and more seasonal occupation [25]. Plant remains mainly cluster in the central part of the Northern Sector and in the southeastern corner of the Main Sector. The central-eastern area, instead, close to the shelter wall, houses the burial area of two individuals where basketry remains could be cautionary interpreted as related to the layout and/or preparation of the burial [26].

**Fig 12:**
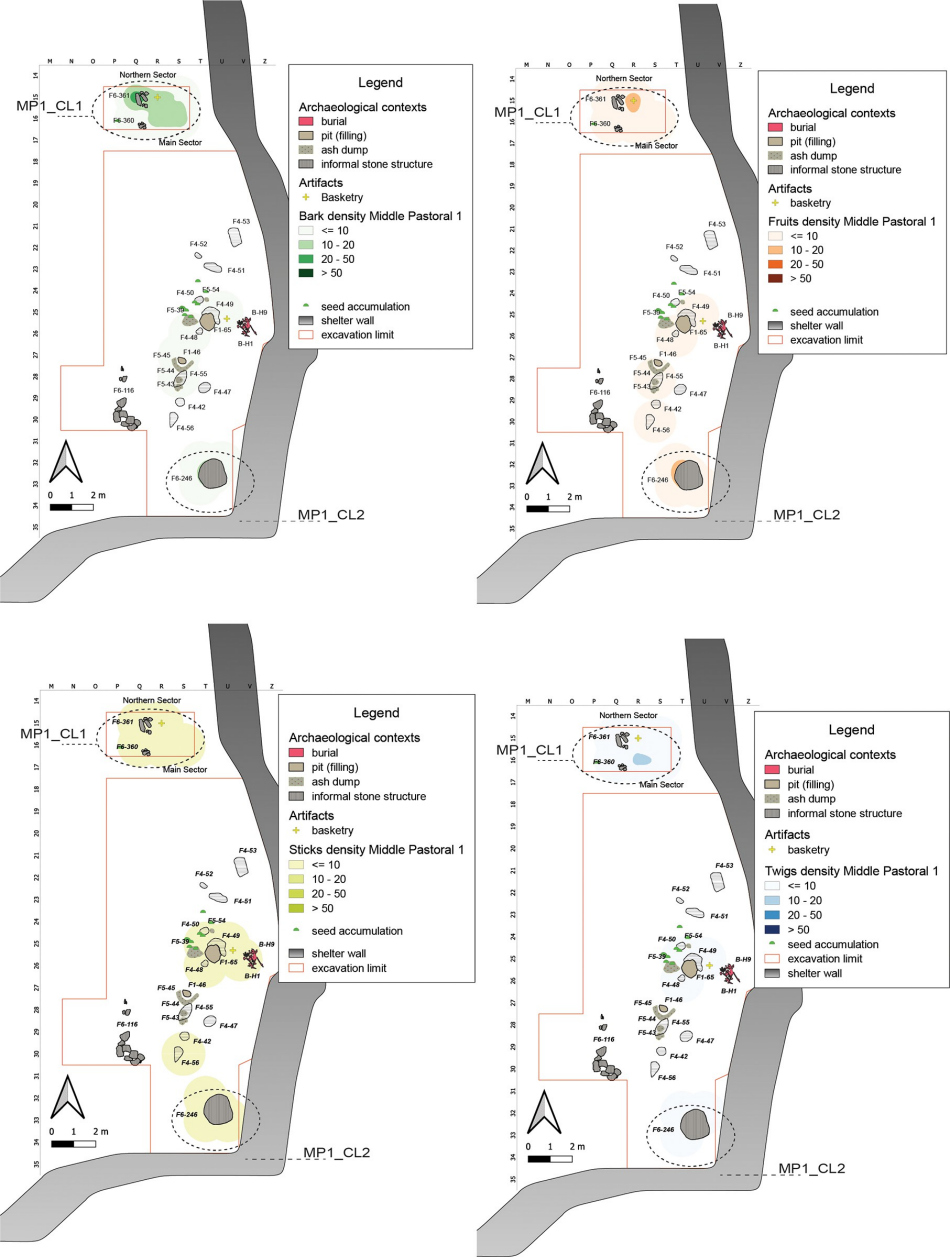
MP1 Kernel Density Estimation. Kernel Density Estimation of selected ecofacts (barks, fruits, sticks and twigs) in MP1, with plotting of relevant artifacts discussed in the text and indication of clusters specified in Table 7.

In the Northern Sector, the area occupied by some stone structures overlaps with the main clusters of plant remains, **MP1_CL1** helping to define a temporary shelter for short-time visitors (Fig 12). In the southeastern portion of the Main Sector, it is possible to identify another area of aggregation, **MP1_CL2**, characterized by the presence of different remains associated with an informal structure: likely an area used for secondary refuse management (Fig 12). The correlation between Sticks and Fruits is slightly higher than that between Sticks and Barks (S3 Table).

#### Middle Pastoral 2 (6400–5600 years cal BP)

**Fig 13:**
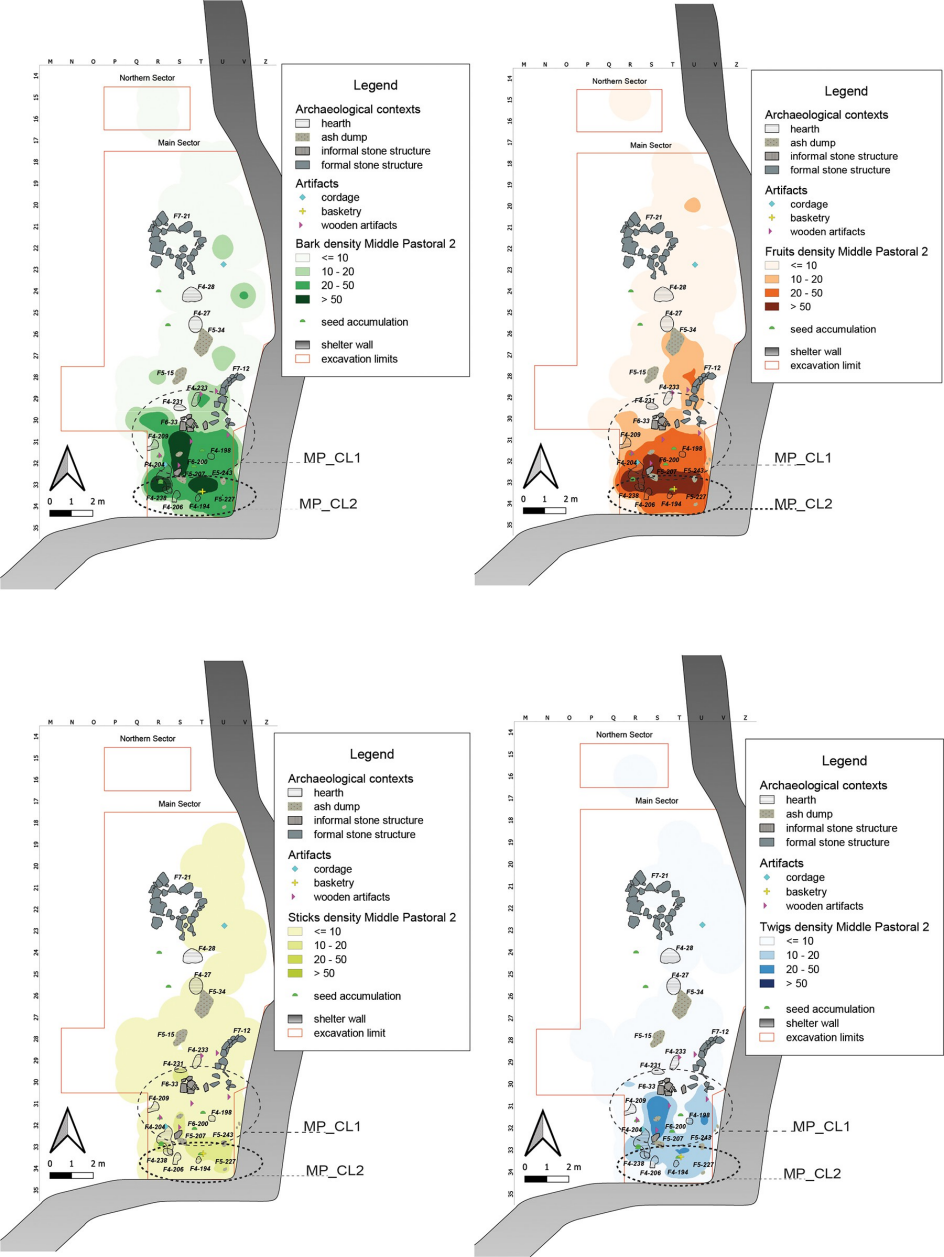
MP2 Kernel Density Estimation. Kernel Density Estimation of selected ecofacts (barks, fruits, sticks and twigs) in MP2, with plotting of relevant artifacts discussed in the text and indication of clusters specified in Table 7.

The shelter in this sub-phase is mainly used in the southern portion of the Main Sector, where numerous hearths and ash accumulations are preserved (Fig 13). Fruits and Barks are here particularly abundant, followed by Twigs and Sticks. Barks and Twigs show a very similar distributional pattern, with higher density toward the outer edge of the area in question, while Fruits tend to be more concentrated along the southern wall of the shelter.

In fact, plant remains are concentrated in the southern area of the Main Sector, **MP2_CL1**, characterized by the presence of numerous hearths and ash accumulations, and a few artefacts on perishable materials (six wooden tools and some basketry remains). Differently from Fruits and Sticks, Barks and Twigs show a density peak immediately south of F6–33, an informal stone structure, also testified by the significant positive correlation between them, much higher than Fruits and Barks (S3 Table) **MP2_CL2**.

In a herding framework, such a distribution in the innermost part of the shelter, between the eastern and southern walls, together with the presence of small hearths interpreted as “corral fires” [99], suggests the presence of an animal management area: the plant remains, such as Barks, Sticks, and Twigs could represent what is left of an enclosure made of perishable material (as also testified by the presence of cordage), whereas the high density of Fruits could be interpreted as forage [66].

### Space and activities during the Late Pastoral

#### Late Pastoral 1 (5900–4300 years cal BP)

**Fig 14:**
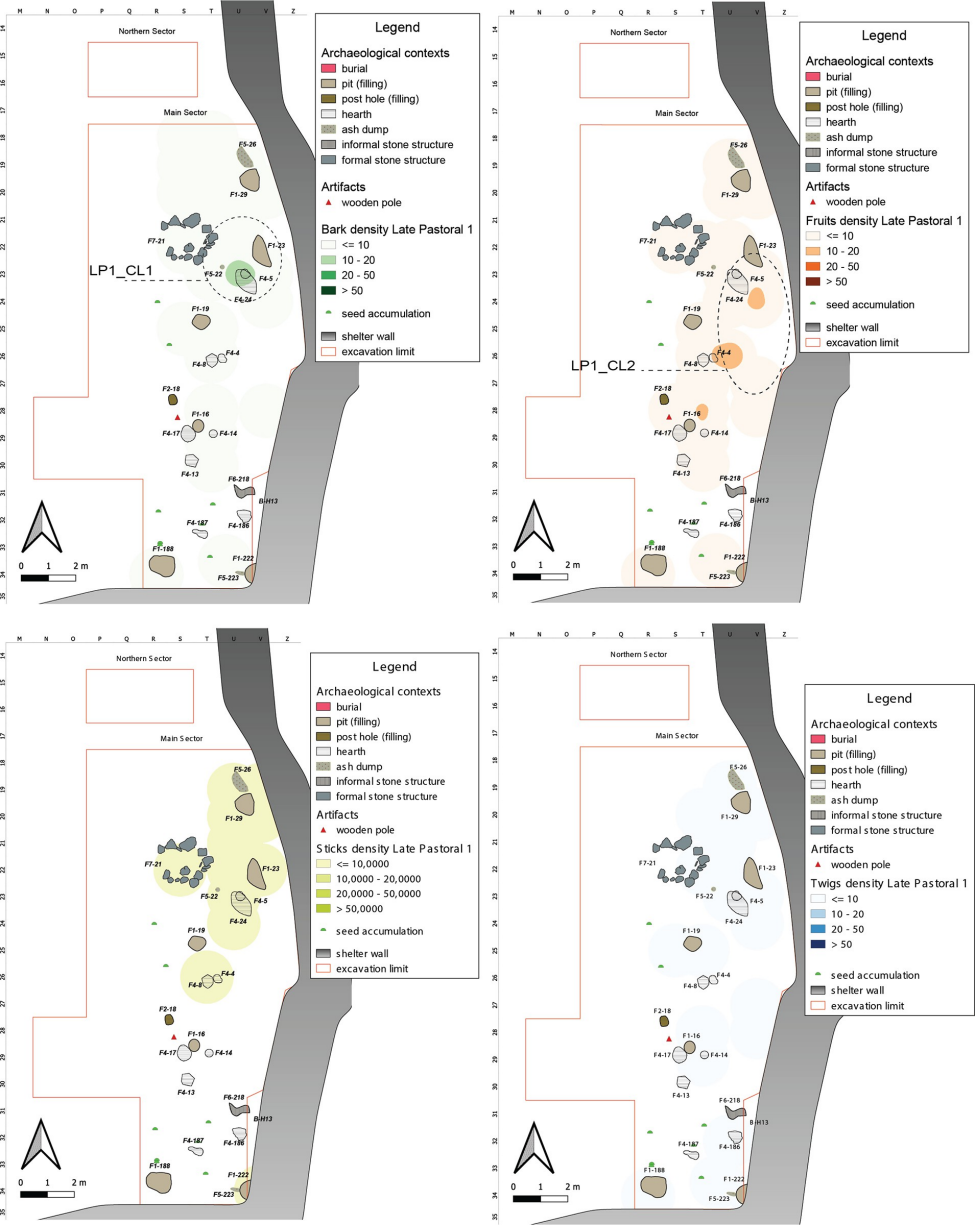
LP1 Kernel Density Estimation. Kernel Density Estimation of selected ecofacts (barks, fruits, sticks and twigs) in LP1, with plotting of relevant artifacts discussed in the text and indication of clusters specified in Table 7.

The Middle Pastoral experiences gradual climatic deterioration, which intensifies significantly in the early stages of the Late Pastoral [100]. In Late Pastoral 1, we observe a substantial reorganization of the settlement system and a shift towards an exclusive reliance on small livestock exploitation for subsistence, mainly goat. This phase is predominantly found in the Main Sector, where plant remains are concentrated towards the shelter’s innermost region, displaying a reasonably varied spatial distribution. However, a common feature is the presence of isolated clusters and a preference for areas adjacent to the shelter’s wall (Fig 14). These isolated clusters correspond to a low correlation index, except for Barks and Twigs (S3 Table).

Within this area, we identify numerous fixtures, including a formal stone structure, four pits, a post hole, and five hearths. This delineates a more organized zone between the shelter wall and the central space. Barks, Sticks, and Twigs tend to cluster more densely in the northeastern part of the Main Sector, gradually diminishing towards the south, LP1_CL1. These materials are often associated with structural elements such as pits, hearths, and ash accumulations, suggesting a multifunctional area encompassing activities like fire-related tasks, storage, and processing.

A study on caprine dung [101] highlights a primary stabling area in the central portion of the Main Sector, near the shelter wall where **LP1_CL2** is located. This may imply the presence of fencing structures, supported by evidence like the existence of a post hole on its outer boundary and a wooden pole. Fruits are also prevalent near the shelter wall, likely resulting from digested fodder, as they are found within dung levels.

In the central-northern zone of the shelter, we observe weaker concentrations of Fruits and Barks. This corresponds to the formal structure F7–21, interpreted as a small installation linked to animal management activities [101].

## General discussion: Plant and site use across the Holocene

The archaeobotanical record at Takarkori shows the biodiversity of wild plants that were distributed in the area in the early to late Holocene. Foragers and herders possessed a deep ecological and environmental knowledge of the environment, as mirrored in the quantity and the careful selection of the different available plant species. The use of plants for the preparation, construction, and maintenance of the living floors and dwelling arrangements are mostly evident during the Late Acacus, and clearly indicates a semi-permanent type of occupation by foragers. This implies a significant investment in time and effort for the realization of facilities.

Spatial configurations reflect a commitment to long-term inhabitation. The intricate relationships and features, characterized by postholes, wooden poles and archaeobotanical remains, along with its consistent use, signifies a substantial investment in establishing a permanent settlement, commencing with the initial habitation in the LA1 and further growing along the LA2 and LA3 subphases. Investing in dwellings has consistently shown a strong link to reduced residential mobility and a deliberate intention for longer stay [102–107]. Additionally, the size and construction investment in huts are directly linked to the expectation of prolonged habitation [108]. The absence of macroremains from the immediate vicinity of structure 131 (LA3) can moreover be interpreted as a result of a specific settlement strategy, contrasting with the evidence of the hut structure 374 in the previous LA2 sub-phase. The latter was likely more temporary and allowed to be dismissed without further “caring”, whereas for the larger, more durable, and stable structure 131, important and “structural” items like poles and side mats might have been deliberately removed as part of an “anticipated mobility” strategy following Kent’s framework [108].

At Takarkori, the site organization layout furthermore points towards a high investment into resource management, as testified by the storage facilities made of basketry and of the differential distribution of ecofacts. Along with the presence of an enclosure for corralling wild animals, it clearly indicates a planned use of various resources. This programming is hence also visible in the systematic and reiterated use of similar spatial layouts in the site, which finds close correlates also in other sites of the region, like Ti-n-Torha East in the northern Tadrart Acacus, where the presence of hut remains are recorded [109, 110], in the remains retrieved from Uan Tabu [65], Uan Afuda and Fozzigiaren [111]. If hence the ways domestic architecture is realized may reflect a “habitus” (*sensu* Bourdieu), the similarity across a sub-region may be indicative of at least a someway unitarian cultural entity. Evidence of residential architecture and increased sedentism was furthermore advanced for the circular outlined structures and floor with leafy remains in the sheltered site of Ti-n-Hanakaten in the Tassili-n-Ajjer in Algeria [64, 112, 113], which further sustain close relationships and cultural affinity between the two areas of Central Sahara [114, 115]. The significant level of reiteration observed in the spatial arrangement at Takarkori over time can be attributed to two possible factors, besides the spectrum of plant species available in the region. Firstly, the presence of material remnants may evoke reminiscences of past occupations, leading to the preservation of previous patterns as part of an active social tradition [49, 53, 116–119]. Alternatively, these material remains may provide favorable conditions that enhance the attractiveness of specific locations within the site for certain activities [94, 102, 120]. Areas designated for waste disposal, for instance, may continue to serve that purpose, while activity areas may be reused for similar or related tasks, facilitating the reuse of debris as recycled materials [15, 56, 121].

Moreover, the spatial redundancy in the site’s layout may also be influenced by strictly functional factors related to the topography and physical characteristics of the location. These factors contribute to meeting the fundamental requirements of dwelling structures, aiming to optimize cost and energy efficiency [117, 122], particularly effective in sheltered locations.

Functionality and maximization in the exploitation of space in terms of accessibility and arrangements are factors that certainly also affected the later occupations of the Pastoral Neolithic. The reconstruction of possible site layout in the Early Pastoral evidence how the concomitant use of the shelter as burial ground in some way influenced the organization of the site. The plant remains associated with the burial H6 might be linked to the layout of the grave installments, as liner or coating in association with the stone arrangements, a feature of this period [26]. The transition to a productive subsistence economy must have led, at least at its beginnings, to a degree of changes in mobility strategies and site organization, as reflected in the decrease of clear and well-defined dwelling installments. The identification of some latent structure as evidenced by plant remains, testifies on the other hand that the site had multiple functions, and was certainly shared by both humans and their livestock.

The presence and the increase of socio-economic importance of animals might be connected to the parallel increase in *Balanites aegyptiaca*, which as testified from different ethnographic sources is a valuable and still used fodder, especially during resting times and when animals are fenced for some period. This can be the case of the agglomerations of desert date fruits in the Middle Pastoral periods, where several elements testify to the co-presence of animals and humans at the site during the time of occupation. These became shorter but repeated and inserted into a scheduled occupation of mountain areas part of a systematic transhumant system of seminomadic herders [100]. Nomadic and highly specialized goat herders characterize the demise of Takarkori’s occupation with short-term and episodic use of use-specific installments, fodder remains and thick dung layers [55, 101].

The results here presented shed further insights into the complex and multifaceted relationships between plant materials and the dwellers of the Takarkori rock shelter. The need to present a diachronic picture of this interconnection hampered the investigations of multiple lines of interpretation and nuances that are evident throughout the millennial occupation of the site. The presence of multiple phases of occupation, which exceeds dozens of generations, implies the equal multiplicity of behaviors that can be reflected within the material and spatial vestiges recognized in the putative living space. Trends and patterns are in fact generalizations that offer us a reconstructed image of reality that can, however, be approximated if interrogated and analyzed with the right degree of formalization and analyticity.

## Final remarks

The archaeobotanical evidence from Takarkori indicates a significant biodiversity of wild plants, reflecting the extensive ecological knowledge of foragers and herders in the early to late Holocene. The complexity and diversity of plant remain assemblages reflect a long history of occupation, spanning multiple generations, and highlight the diverse and evolving nature of human-environment interactions at the site evidencing:

- The careful selection and use of plants for different purposes, such as construction materials for living floors and dwellings, suggest a deep knowledge of local ecology and vegetation and a significant investment in semi-permanent structures during the Late Acacus period.
- The consistent presence of certain plant species across different occupation phases reflects a systematic approach to resource management, including the use of specific plants for food, fodder, and other utilitarian purposes.
- The spatial distribution of plant remains, including their association with specific structures and activity areas, indicates deliberate planning in the use of plant resources, such as the use of *Balanites aegyptiaca* for animal fodder during the Middle Pastoral period.
- The evidence of plant materials in burial contexts and the presence of leafy remains in sheltered areas highlight the diverse uses of plants beyond subsistence, including ritual and cultural practices.

## Supporting information

S1 Fig Monte Carlo test of spatial randomness for the point pattern of selected ecofacts. Monte Carlo test of spatial randomness for the point pattern (Ripley’s K function) of selected ecofact categories, according to sub-phases (from the oldest LA1, top left, to the youngest LP1, bottom right). Observed K-function is on y axes (black line) compared to the expected randomly distributed points (red dotted line) in the expected range of 95% confidence envelopes (gray area) for the hypothesis of complete spatial randomness, obtained from 100 independent randomizations.
(TIF)

S1 Table Percentage of selected plant remains. The percentage of selected plant remains, by ACs (in brackets the absolute frequency) and chronological sub-phases.
(DOCX)

S2 Table QCT results for the archaeobotanical selected samples by sub-phase. (DOCX)

S3 Table Pearson’s correlation coefficient *r* between selected ecofacts by chronological sub-phase. (DOCX)

S1 File (XLSX)
