# Supplementary figures and images for: Spatial analyses of archaeobotanical record reveal site uses and activities at Early to Middle Holocene Takarkori (Libya, Central Sahara)
Source: PLoS One. 2024 Oct 23;19(10):e0310739. doi: 10.1371/journal.pone.0310739 (PMC11498675; doi:10.1371/journal.pone.0310739)

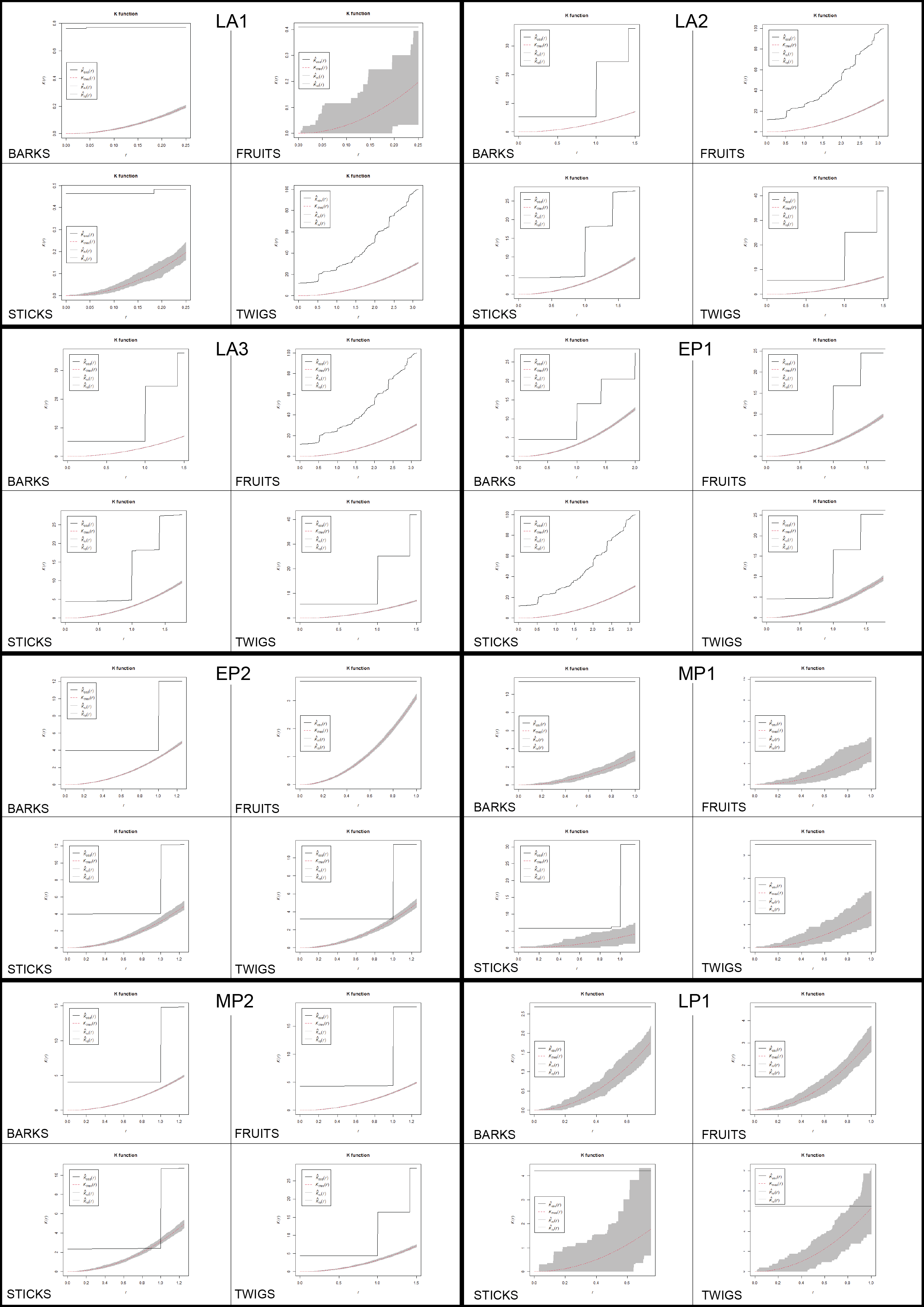

Supplement: S1 Fig — Monte Carlo test of spatial randomness for the point pattern (Ripley’s K function) of selected ecofact categories, according to sub-phases (from the oldest LA1, top left, to the youngest LP1, bottom right). Observed K-function is on y axes (black line) compared to the expected randomly distributed points (red dotted line) in the expected range of 95% confidence envelopes (gray area) for the hypothesis of complete spatial randomness, obtained from 100 independent randomizations. (TIF) [file pone.0310739.s001.tif]
