# Supplementary material for: Spatial analyses of archaeobotanical record reveal site uses and activities at Early to Middle Holocene Takarkori (Libya, Central Sahara)
Source: PLoS One. 2024 Oct 23;19(10):e0310739. doi: 10.1371/journal.pone.0310739 (PMC11498675; doi:10.1371/journal.pone.0310739)
Supplement: S1 Table — The percentage of selected plant remains, by ACs (in brackets the absolute frequency) and chronological sub-phases. (DOCX) [file pone.0310739.s002.docx]

**S1 Table**

| **Sub-phase** | **Class/ category** | **M1** | **M2** | **M3** | **M4** | **M5** | **M6** | **M7** | **M8** | **F1** | **F4** | **F5** | **F6** | **F7** | **B** |
| --- | --- | --- | --- | --- | --- | --- | --- | --- | --- | --- | --- | --- | --- | --- | --- |
| LA1 | barks (323) | 11.8 | - | - | 61.0 | - | - | - | 0.3 | - | 1.2 | 0.6 | - | 25.1 | - |
|  | fruits (19) | 5.3 | - | - | 89.5 | - | - | - | - | - | 5.3 | - | - | - | - |
|  | sticks (79) | 8.9 | - | - | 72.2 | - | - | - | 1.3 | - | 15.2 | - | - | 2.5 | - |
|  | twigs (117) | 17.9 | - | - | 69.2 | - | - | - | - | - | 7.7 | 5.1 | - | - | - |
|  | *Average, by ACs* | *12.5* | *-* | *-* | *65.4* | *-* | *-* | *-* | *0.4* | *-* | *4.8* | *1.5* | *-* | *15.4* | - |
| LA2 | barks (1565) | - | - | - | 46.2 | 1.5 | 8.4 | - | 19.7 | - | 15.1 | - | - | 8.8 | - |
|  | fruits (178) | - | - | - | 57.9 | 6.7 | 2.2 | - | 15.2 | - | 15.2 | - | - | 2.8 | - |
|  | sticks (587) | - | - | - | 48.0 | 2.6 | 4.4 | - | 21.8 | - | 19.8 | - | - | 3.1 | - |
|  | twigs (503) | - | - | - | 50.3 | 1.2 | 7.2 | - | 24.1 | - | 13.3 | - | - | 3.8 | - |
|  | *Average, by ACs* | - | *-* | *-* | *48.0* | *2.0* | *7.0* | *-* | *20.7* | *-* | *15.8* | - | - | *6.3* | *-* |
| LA3 | barks (1113) | - | - | - | 74.3 | - | 2.2 | - | 3.3 | - | 5.2 | 10.7 | - | 3.3 | - |
|  | fruits (452) | - | - | - | 46.9 | - | 3.1 | - | 4.6 | - | 14.2 | 30.8 | - | 0.4 | - |
|  | sticks (514) | - | - | - | 66.3 | - | 0.8 | - | 4.1 | - | 8.8 | 18.3 | - | 1.2 | - |
|  | twigs (475) | - | - | - | 67.4 | - | 2.3 | - | 6.5 | - | 8.8 | 13.5 | - | 0.8 | - |
|  | *Average, by ACs* | - | *-* | *-* | *66.6* | *-* | *2.1* | *-* | *4.3* | *-* | *8.2* | *16.3* | *-* | *1.9* | *-* |
| EP1 | barks (525) | - | 46.1 | 0.4 | 12.8 | 0.2 | - | 5.3 | 12.6 | - | 3.2 | - | - | 19.2 | - |
|  | fruits (414) | - | 78.8 | 1.2 | 7.2 | 3.1 | - | 0.6 | 3.9 | - | 1.9 | - | - | 2.7 | - |
|  | sticks (223) | - | 71.3 | 4.9 | 8.1 | 0.4 | - | 3.6 | 7.2 | - | 1.8 | - | - | 2.7 | - |
|  | twigs (305) | - | 64.3 | 0.3 | 13.8 | 0.7 | - | 4.9 | 4.6 | - | 3.0 | - | - | 6.9 | - |
|  | *Average, by ACs* | - | *62.6* | *1.3* | *10.7* | *1.2* | - | *3.7* | *7.6* | *-* | *2.6* | - | - | *9.5* | *-* |
| EP2 | barks (675) | - | - | 0.6 | 94.5 | - | - | 0.3 | - | 2.5 | 0.7 | 1.3 | - | - | - |
|  | fruits (482) | - | - | 1.7 | 84.6 | - | - | 1.0 | 0.8 | 1.9 | 1.7 | 8.3 | - | - | - |
|  | sticks (172) | - | - | 1.7 | 86.6 | - | - | - | - | 1.7 | 2.3 | 7.6 | - | - | - |
|  | twigs (210) | - | - | 1.4 | 89.0 | - | - | - | 0.5 | 0.8 | 4.8 | 3.3 | - | - | - |
|  | *Average, by ACs* | *-* | - | *1.2* | *89.8* | *-* | - | *0.4* | *0.3* | *2.0* | *1.7* | *4.5* | *-* | - | - |
| MP1 | barks (117) | - | - | - | 63.2 | - | - | - | - | 8.5 | 0.9 | 1.7 | 25.6 | - | - |
|  | fruits (68) | - | - | - | 42.0 | - | - | - | - | 1.4 | 4.3 | 1.4 | 50.7 | - | - |
|  | sticks (35) | - | - | - | 40.0 | - | - | - | - | 5.7 | 8.6 | - | 42.9 | - | 2.9 |
|  | twigs (43) | - | - | - | 69.8 | - | - | - | - | 2.3 | 4.7 | - | 23.3 | - | - |
|  | *Average, by ACs* | - | - | - | *55.7* | *-* | - | - | - | *5.3* | *3.4* | *1.1* | *34.1* | - | *0.4* |
| MP2 | barks (886) | - | - | - | 98.1 | - | - | - | - | - | 0.9 | 1.0 | - | - | - |
|  | fruits (888) | - | - | - | 94.9 | - | - | - | - | - | 1.4 | 3.7 | - | - | - |
|  | sticks (211) | - | - | - | 91.5 | - | - | - | - | - | 4.7 | 3.3 | 0.5 | - | - |
|  | twigs (314) | - | - | - | 97.1 | - | - | - | - | - | 0.6 | 2.2 | - | - | - |
|  | *Average, by ACs* | - | *-* | - | *96.1* | - | - | - | - | - | *1.4* | *2.4* | *0.4* | - | - |
| LP1 | barks (109) | - | - | - | 51.4 | - | - | 8.3 | - | 11.9 | 16.5 | 0.9 | - | 11.0 | - |
|  | fruits (118) | - | - | - | 31.4 | - | - | 27.1 | - | 18.6 | 3.4 | 4.2 | - | 15.3 | - |
|  | sticks (23) | - | - | - | 26.1 | - | - | - | - | 21.7 | 26.1 | 4.3 | - | 21.7 | - |
|  | twigs (48) | - | - | - | 39.6 | - | - | 12.5 | - | 10.4 | 18.8 | 6.3 | - | 12.5 | - |
|  | *Average, by ACs* | - | - | - | *39.6* | - | - | *15.8* | - | *15.1* | *12.4* | *3.4* | - | *13.8* | - |
