# Supplementary material for: Spatial analyses of archaeobotanical record reveal site uses and activities at Early to Middle Holocene Takarkori (Libya, Central Sahara)
Source: PLoS One. 2024 Oct 23;19(10):e0310739. doi: 10.1371/journal.pone.0310739 (PMC11498675; doi:10.1371/journal.pone.0310739)
Supplement: S2 Table — (DOCX) [file pone.0310739.s003.docx]

**S2 Table**

| **SUBPHASE** | **Material Class** | **X2** | **DF** | **P VALUE** | **ALTERN. HYPOTH** |
| --- | --- | --- | --- | --- | --- |
| LP1 | BARKS | 165.11 | 24 | <2.e-16 | two.sided |
|  | FRUITS | 208.16 | 24 | <2.e-16 | two.sided |
|  | STICKS | 99.826 | 24 | 6.433E-08 | two.sided |
|  | TWIGS | 77 | 24 | <3.569e-07 | two.sided |
| MP2 | BARKS | 1943.3 | 24 | < 2.2e-16 | two.sided |
|  | FRUITS | 2074 | 24 | < 2.2e-16 | two.sided |
|  | STICKS | 328.2 | 24 | < 2.2e-16 | two.sided |
|  | TWIGS | 776,84 | 24 | < 2.2e-16 | two.sided |
| MP1 | BARKS | 444.99 | 24 | < 2.2e-16 | two.sided |
|  | FRUITS | 251.51 | 24 | < 2.2e-16 | two.sided |
|  | STICKS | 93.429 | 24 | 7.75E-07 | two.sided |
|  | TWIGS | 191.3 | 24 | < 2.2e-16 | two.sided |
| EP2 | BARKS | 1263.3 | 24 | < 2.2e-16 | two.sided |
|  | FRUITS | 992.48 | 24 | < 2.2e-16 | two.sided |
|  | STICKS | 343.12 | 24 | < 2.2e-16 | two.sided |
|  | TWIGS | 341.09 | 24 | < 2.2e-16 | two.sided |
| EP1 | BARKS | 553.68 | 24 | < 2.2e-16 | two.sided |
|  | FRUITS | 875.21 | 24 | < 2.2e-16 | two.sided |
|  | STICKS | 499.07 | 24 | < 2.2e-16 | two.sided |
|  | TWIGS | 243.25 | 24 | < 2.2e-16 | two.sided |
| LA3 | BARKS | 1082.9 | 24 | < 2.2e-16 | two.sided |
|  | FRUITS | 441.87 | 24 | < 2.2e-16 | two.sided |
|  | STICKS | 952.67 | 24 | < 2.2e-16 | two.sided |
|  | TWIGS | 407.48 | 24 | < 2.2e-16 | two.sided |
| LA2 | BARKS | 3996.5 | 24 | < 2.2e-16 | two.sided |
|  | FRUITS | 405.18 | 24 | < 2.2e-16 | two.sided |
|  | STICKS | 1134 | 24 | < 2.2e-16 | two.sided |
|  | TWIGS | 1433.3 | 24 | < 2.2e-16 | two.sided |
| LA1 | BARKS | 1297.8 | 24 | < 2.2e-16 | two.sided |
|  | FRUITS | 66.526 | 24 | < 2.2e-16 | two.sided |
|  | STICKS | 220.5 | 24 | <2.2e-16 | two.sided |
|  | TWIGS | 270.39 | 24 | <2.2e-16 | two.sided |
