# Supplementary material for: Spatial analyses of archaeobotanical record reveal site uses and activities at Early to Middle Holocene Takarkori (Libya, Central Sahara)
Source: PLoS One. 2024 Oct 23;19(10):e0310739. doi: 10.1371/journal.pone.0310739 (PMC11498675; doi:10.1371/journal.pone.0310739)
Supplement: S3 Table — (DOCX) [file pone.0310739.s004.docx]

**S3 Table**

| Sub-phases | Ecofacts | | | | |
| --- | --- | --- | --- | --- | --- |
| LP1 |  | **barks** | **twigs** | **sticks** | **fruits** |
|  | **barks** | 1 | 0.591061 | 0.33082 | 0.113381 |
|  | **twigs** | 0.591061 | 1 | 0.087147 | -0.01062 |
|  | **sticks** | 0.33082 | 0.087147 | 1 | 0.025521 |
|  | **fruits** | 0.113381 | -0.01062 | 0.025521 | 1 |
| MP2 |  | **barks** | **twigs** | **sticks** | **fruits** |
|  | **barks** | 1 | 0.782157 | 0.298889 | 0.47331 |
|  | **twigs** | 0.782157 | 1 | 0.256394 | 0.374504 |
|  | **sticks** | 0.298889 | 0.256394 | 1 | 0.324457 |
|  | **fruits** | 0.47331 | 0.374504 | 0.324457 | 1 |
| MP1 |  | **barks** | **twigs** | **sticks** | **fruits** |
|  | **barks** | 1 | 0.409801 | 0.537179 | 0.155032 |
|  | **twigs** | 0.409801 | 1 | 0.120697 | 0.059492 |
|  | **sticks** | 0.537179 | 0.120697 | 1 | 0.570398 |
|  | **fruits** | 0.155032 | 0.059492 | 0.570398 | 1 |
| EP2 |  | **barks** | **twigs** | **sticks** | **fruits** |
|  | **barks** | 1 | 0.533563 | 0.359998 | 0.376982 |
|  | **twigs** | 0.533563 | 1 | 0.22212 | 0.251004 |
|  | **sticks** | 0.359998 | 0.22212 | 1 | 0.336075 |
|  | **fruits** | 0.376982 | 0.251004 | 0.336075 | 1 |
| EP1 |  | **barks** | **twigs** | **sticks** | **fruits** |
|  | **barks** | 1 | 0.417813 | 0.210588 | 0.113426 |
|  | **twigs** | 0.417813 | 1 | 0.265301 | 0.208605 |
|  | **sticks** | 0.210588 | 0.265301 | 1 | 0.266688 |
|  | **fruits** | 0.113426 | 0.208605 | 0.266688 | 1 |
| LA3 |  | **barks** | **twigs** | **sticks** | **fruits** |
|  | **barks** | 1 | 0.492939 | 0.520277 | 0.241762 |
|  | **twigs** | 0.492939 | 1 | 0.593777 | 0.243879 |
|  | **sticks** | 0.520277 | 0.593777 | 1 | 0.409481 |
|  | **fruits** | 0.241762 | 0.243879 | 0.409481 | 1 |
| LA2 |  | **barks** | **twigs** | **sticks** | **fruits** |
|  | **barks** | 1 | 0.521676 | 0.702482 | 0.414401 |
|  | **twigs** | 0.521676 | 1 | 0.503189 | 0.232858 |
|  | **sticks** | 0.702482 | 0.503189 | 1 | 0.335494 |
|  | **fruits** | 0.414401 | 0.232858 | 0.335494 | 1 |
| LA1 |  | **barks** | **twigs** | **sticks** | **fruits** |
|  | **barks** | 1 | 0.03853 | 0.164329 | -0.05982 |
|  | **twigs** | 0.03853 | 1 | 0.195933 | -0.04972 |
|  | **sticks** | 0.164329 | 0.195933 | 1 | -0.22442 |
|  | **fruits** | -0.05982 | -0.04972 | -0.22442 | 1 |
